# New Insights into the Reaction Paths of Hydroxyl Radicals with Purine Moieties in DNA and Double-Stranded Oligodeoxynucleotides

**DOI:** 10.3390/molecules24213860

**Published:** 2019-10-26

**Authors:** Chryssostomos Chatgilialoglu, Marios G. Krokidis, Annalisa Masi, Sebastian Barata-Vallejo, Carla Ferreri, Michael A. Terzidis, Tomasz Szreder, Krzysztof Bobrowski

**Affiliations:** 1 Istituto per la Sintesi Organica e la Fotoreattività, Consiglio Nazionale delle Ricerche, 40129 Bologna, Italy; m.krokidis@inn.demokritos.gr (M.G.K.); annalisa.masi@isof.cnr.it (A.M.); carla.ferreri@isof.cnr.it (C.F.); mterzidi@gmail.com (M.A.T.); 2 Center for Advanced Technologies, Adam Mickiewicz University, 61-614 Poznań, Poland; 3 Institute of Nanoscience and Nanotechnology, N.C.S.R. “Demokritos”, 15310 Agia Paraskevi Attikis, Greece; 4 Departamento de Quimíca Organíca, Facultad de Farmacia y Bioquimíca, Universidad de Buenos Aires, Junin 954, Buenos Aires CP 1113, Argentina; 5 Centre of Radiation Research and Technology, Institute of Nuclear Chemistry and Technology, Dorodna 16, 03-195 Warsaw, Poland; t.szreder@ichtj.waw.pl

**Keywords:** DNA damage, 5′,8-cyclopurines, 8-oxo-dG, free radicals, pulse radiolysis, gamma radiolysis, Fenton reaction, oligonucleotides

## Abstract

The reaction of hydroxyl radical (HO^•^) with DNA produces many primary reactive species and many lesions as final products. In this study, we have examined the optical spectra of intermediate species derived from the reaction of HO^•^ with a variety of single- and double-stranded oligodeoxynucleotides and ct-DNA in the range of 1 μs to 1 ms by pulse radiolysis using an Intensified Charged Coupled Device (ICCD) camera. Moreover, we applied our published analytical protocol based on an LC-MS/MS system with isotopomeric internal standards to enable accurate and precise measurements of purine lesion formation. In particular, the simultaneous measurement of the four purine 5′,8-cyclo-2′-deoxynucleosides (cPu) and two 8-oxo-7,8-dihydro-2′-deoxypurine (8-oxo-Pu) was obtained upon reaction of genetic material with HO^•^ radicals generated either by γ-radiolysis or Fenton-type reactions. Our results contributed to the debate in the literature regarding absolute level of lesions, method of HO^•^ radical generation, 5′*R*/5′*S* diastereomeric ratio in cPu, and relative abundance between cPu and 8-oxo-Pu.

## 1. Introduction

**Figure 1:**
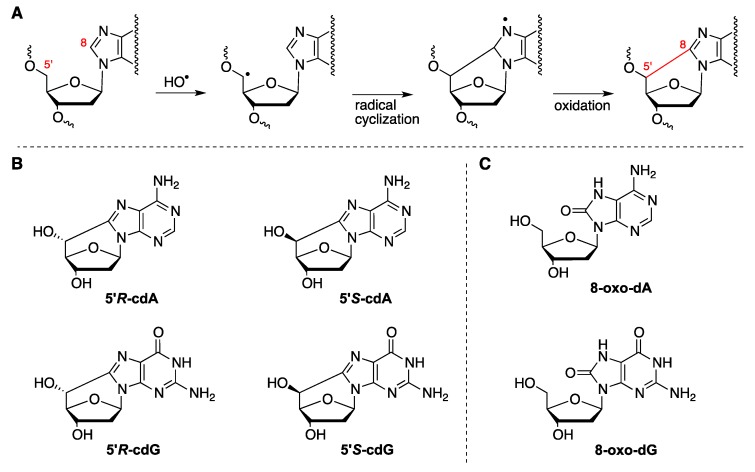
(**A**) purine 2′-deoxynucleoside reacts with hydroxyl radical (HO^•^) yielding the purine 5′,8-cyclo-2′-deoxynucleoside (cPu) via cyclization of C5′ radical followed by oxidation; (**B**) structures of 5′,8-cyclo-2′-deoxyadenosine (cdA) and 5′,8-cyclo-2′-deoxyguanosine (cdG) in their 5′*R* and 5′*S* diastereomeric forms; (**C**) structures of 8-oxo-2′-deoxyadenosine (8-oxo-dA) and 8-oxo-2′-deoxyguanosine (8-oxo-dG).

Hydroxyl radicals (HO^•^) are highly reactive with many compounds and DNA is not an exception. Indeed, HO^•^ radicals are known for their reactivity and ability to cause chemical modifications to DNA, the site of attack being both the base moieties (85–90%) and the 2-deoxyribose units [1,2]. The attack at H5′ of DNA by HO^•^ radicals is estimated to be 55% of all possible sugar positions and the resulting C5′ radical in the purine nucleotide moieties leads to the formation of purine 5′,8-cyclo-2′-deoxynucleosides (cPu) as final products (Figure 1A) [3,4]. The 5′,8-cyclo-2′-deoxyadenosine (cdA) and 5′,8-cyclo-2′-deoxyguanosine (cdG) exist in 5′*R* and 5′*S* diastereoisomeric forms (Figure 1B). These tandem-type lesions, generated by the attack of HO^•^ radicals or direct irradiation damage [5], have been identified in mammalian cellular DNA in vivo [6,7,8,9,10] and are substrates of nucleotide excision repair (NER) [4,11,12]. On the other hand, the addition of HO^•^ radicals to the guanine and adenine moieties affords a variety of products including the well-known 8-oxo-7,8-dihydro-2′-deoxyadenosine (8-oxo-dA) and 8-oxo-7,8-dihydro-2′-deoxyguanosine (8-oxo-dG) lesions (Figure 1C) [13]. Like HO^•^ radicals, other oxidizing species such as H_2_O_2_, singlet oxygen or ONOO^−^ are able to generate 8-oxo-Pu lesions that are removed by the base excision repair (BER) system [14].

Attempts to accurately determine the level of the four cPu lesions in DNA are numerous [4,15,16]. A detailed protocol for the simultaneous quantification of the four cPu lesions and two 8-oxo-Pu of DNA has also been provided by some of us [4,16]. Liquid chromatography-tandem mass spectrometry (LC-ESI-MS/MS) analysis, following a top–down approach starting from the genetic material and going down to a single nucleoside level, establishes accurate quantification of these lesions. The use of isotopically labeled reference compounds for the lesions further enhances the reliability of the process, increasing to a great extent the reproducibility and the recovery of the quantification protocol.

Pulse radiolysis studies on the reaction of HO^•^ radicals with DNA and its model systems (oligonucleotides) are limited due to the cost of starting material. In the present work, we explored the Intensified Charge-Coupled Device (ICCD) as an alternative of photomultiplier (PMT) for transient spectra measurements [17]. The possibility to record the transient spectra using the ICCD camera has the advantage of reduced sample size. Such an approach has been already successfully applied for pulse radiolysis studies on the reaction of HO^•^ radicals with the calcium-saturated forms of wild-type calmodulin and its Met-deficient mutant [18].

The objective of this work is dual: (i) to gain information on the optical absorption of transient spectra of the reaction of HO^•^ radical with DNA and its model systems by pulse radiolysis, and (ii) to apply our protocol based on the stable isotope-dilution tandem mass spectrometry technique for the quantification of HO^•^ radical induced cPu and 8-oxo-Pu lesions within DNA and its model systems in gamma-irradiated aqueous solutions or Fenton-type reactions. In principle, the two techniques employed (pulse radiolysis of transient spectra in the range of 1 μs to 1 ms and LC-ESI-MS/MS for product identification) complement each other, that is, acquired information of the intermediate reactive species that lead to the observed stable products on the same material when exposed to HO^•^ radicals. In order to achieve our aims, we examined the reactivity of HO^•^ radical with calf-thymus DNA (ct-DNA) and a variety of single-stranded (ss) or doubled-stranded (ds) oligodeoxynucleotides (ODNs), which are the simplest biomimetic models of DNA that respect the biological characteristics (Table 1).

## 2. Results and Discussion

### 2.1. Radiolytic Production of Transients

Radiolysis of neutral water leads to the reactive species e_aq_^−^, HO^•^ and H^•^, as shown in Reaction 1, together with H^+^ and H_2_O_2_. The values in parentheses represent the radiation chemical yields (*G*) in units of μmol J^−1^. In N_2_O-saturated solution (~0.02 M of N_2_O), e_aq_^−^ are converted into HO^•^ radical via Reaction 2 (*k*_2_ = 9.1 × 10^9^ M^−1^ s^−1^), with *G*(HO^•^) = 0.55 μmol J^−1^, i.e., HO^•^ radicals and H^•^ atoms account for 90 and 10%, respectively, of the reactive species [19,20]. The rate constants for the reactions of HO^•^ radicals and H^•^ atoms with DNA (Reactions 3 and 4) have been reported to be ca. 2.5 × 10^8^ M^−1^ s^−1^ and 6 × 10^7^ M^−1^ s^−1^, respectively [19,20]:

H_2_O + *ɣ-irr*/e-beam → e_aq_^−^(0.27), HO^•^(0.28), H^•^(0.062), (1)

e_aq_^−^ + N_2_O + H_2_O → HO^•^ + N_2_ + HO^−^, (2)

HO^•^ + DNA (or ODN) → radical product, (3)

H^•^ + DNA (or ODN) → radical product. (4)

### 2.2. Pulse Radiolysis in Aqueous Solutions

Pulse radiolysis is a time-resolved technique that gives an opportunity to look into very short time domains. Therefore, it allows detection and spectral/kinetic characterization of very short-lived transients like radicals, radical-ions, and excited states. In a typical experiment, the UV-Vis spectral changes obtained from the pulse irradiation of N_2_O-saturated solution containing ca. 1 mM of nucleoside are monitored. The possibility to record the transient spectra using the ICCD camera has a great advantage over PMT since it allows to work with extremely valuable micro-volume liquid samples.

#### 2.2.1. Comparison of PMT and ICCD Detection Methods Using Nucleosides

In order to check first reliability of the ICCD camera, the transient spectra resulted from the reactions of HO^•^ radicals with single nucleosides (dC, dG, T, and dA) were recorded by the PMT and ICCD camera, and then the two spectra obtained were compared.

**Figure 2:**
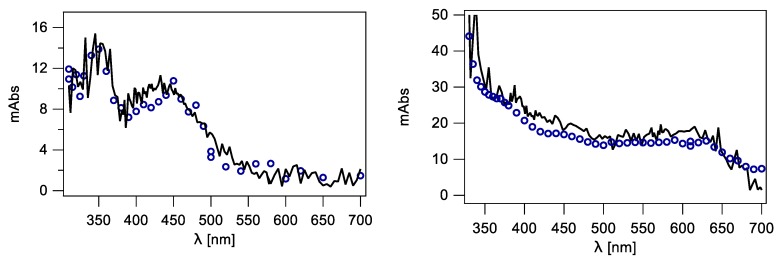
Transient absorption spectra recorded using PMT (**◯**) and ICCD (**−**); (Left) 2 μs after electron pulse in N_2_O-saturated phosphate buffered (50 mM) aqueous solution containing 1 mM 2′-deoxycytidine (dC); (Right) 1 μs after electron pulse in N_2_O-saturated phosphate buffered (50 mM) aqueous solution containing 1 mM 2′-deoxyguanosine (dG), at pH 7.

**Figure 3:**
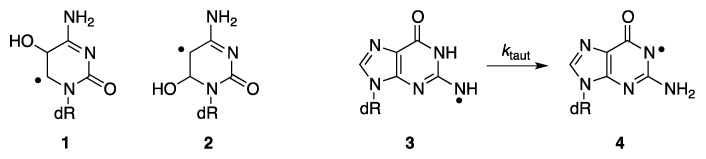
The main species generated by the reaction of HO^•^ radical with dC and dG, respectively.

The spectral changes obtained by the two detection systems after pulse irradiation of a N_2_O-saturated sodium phosphate 50 mM, pH 7, solution of 1 mM 2′-deoxycytidine (representing pyrimidine derivative) superimpose more than satisfactorily and are shown in Figure 2 (left panel). The optical absorption spectra taken 2 μs after the pulse are characterized by two distinctive absorption bands with λ_max_ ~350 and 440 nm. The present results are in agreement with those from the literature [21,22]. This spectrum is mainly due to 5-OH-6-yl radical **1** formed by addition at C5 with ~87% yield, 6-OH-5-yl radicals **2** formed by addition at C6 (Figure 3) and with some minor contribution of H^•^-adduct radicals. The H-atom abstraction from the sugar moiety can also be considered; however, the resulting radicals do not absorb significantly in the wavelength region of interest.

Similarly, the spectral changes obtained by the two detection systems for 2′-deoxyguanosine (purine representative) superimpose satisfactorily and are shown in Figure 2 (right panel). The optical absorption spectra taken 1 μs after the pulse are characterized by a broad absorption band with a weakly marked maximum at λ_max_ ~610 nm. This absorption band was earlier assigned to a guanyl radical **3** (Figure 3) formed by hydrogen abstraction from the exocyclic NH_2_ with ~65% yields, which undergoes further a water-assisted tautomerization to the most stable isomer **4** with a *k*_taut_ = 2.3 × 10^4^ s^−1^ [23,24,25]. Moreover, the absorption spectra obtained are in agreement with those reported in the literature. The spectrum in the range 400–600 nm is flat without a clearly pronounced maximum. Contribution of 8-hydroxyl radical adduct to the absorption spectrum in the short wavelength range (<400 nm) also has to be taken into account [23,25].

#### 2.2.2. Mixture of dC and dG Nucleosides

**Figure 4:**
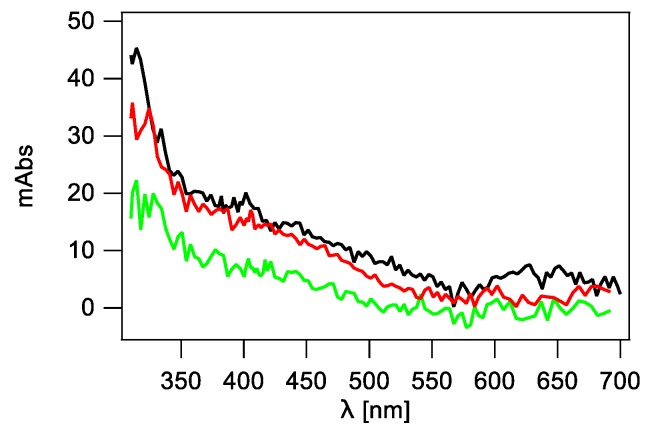
Transient absorption spectra recorded using ICCD (**−**) 1 μs, (**−**) 50 μs, and (**−**) 1 ms after electron pulse in N_2_O-saturated phosphate buffered (50 mM) aqueous solutions containing mixture of 2′-deoxyguanosine (dG, 0.5 mM) and 2′-deoxycytidine (dC, 0.5 mM) with concentration ratio 1:1 at pH 7.

With this information in hand, the transient absorption spectra resulted from the reaction of HO^•^ radicals with the mixture of dG and dC present in an aqueous solution in a concentration ratio of 1:1 were recorded only by an ICCD camera on the time domain between 1 μs to 1 ms (Figure 4). The optical absorption spectrum recorded 1 μs after the pulse is characterized by a broad absorption band in the region of 600–650 nm, a distinctive shoulder in the region of 350–400 nm and a sharp absorption band with λ_max_ ~310 nm. These spectral characteristics are consistent with the presence of transients derived from dG and dC. Moreover, absorption intensity of the 610 nm band is nearly half of the absorption intensity measured in the solution containing only 2′-deoxyguanosine (Figure 2, right panel). This observation is not surprising taking into account equal concentrations of dG and dC nucleosides and their respective rate constants with HO^•^ radicals which are very similar, and equal to (5.7 ± 0.4) × 10^9^ M^−1^s^−1^ [24], and (6.0 ± 1.5) × 10^9^ M^−1^s^−1^ [21], respectively.

#### 2.2.3. Mixture of dC, dG, T and dA Nucleosides vs. Calf-Thymus DNA (ct-DNA)

The subsequent chemical system subjected to irradiation was a phosphate buffered (50 mM) aqueous solution containing a mixture of four nucleosides: dC, dG, T (thymidine) and dA (2′-deoxyadenosine) in the following concentration ratios. The first pair of nucleosides (dC and dG) and the second pair of nucleosides (T and dA) were present in a concentration ratio 1:1. In turn, the two respective pairs of nucleosides were present in a concentration ratio 2:3, which mimics the ratio of these nucleosides present in ct-DNA.

**Figure 5:**
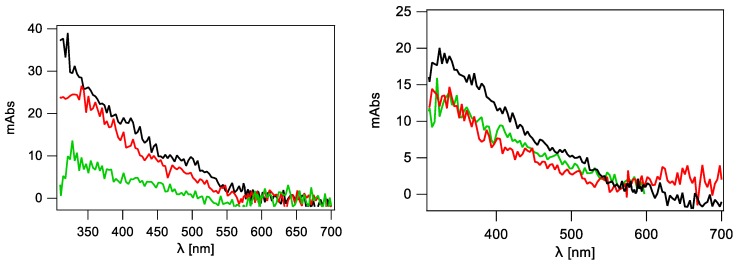
Transient absorption spectra recorded using ICCD in N_2_O-saturated phosphate buffered (50 mM) aqueous solutions containing (left panel) a mixture of four nucleosides (see text) and (right panel) ct-DNA, at natural pH: (−) 1 μs, (**−**) 50 μs, and (**−**) 1 ms after electron pulse.

The optical absorption spectrum recorded 1 μs after the pulse in an aqueous buffered solution containing a mixture of four nucleosides (dC, dG, T and dA) (Figure 5, left panel) is similar to that observed in solution with a mixture of two nucleosides (dC and dG, see Figure 4) except two features: the absorption band in the region >600 nm is absent while two distinctive shoulders in the region 460–500 nm and 400–420 nm appear. The first feature can be rationalized by taking into account the concentration ratio of nucleosides present in the solution as 2:2:3:3 and their respective rate constants with HO^•^ radicals which are nearly equal [2]. Taking a simple competition kinetics of these four nucleosides for HO^•^ radicals, one can easily calculate that at most 20% of all available HO^•^ radicals can react with dG and give rise to the guanyl-type radicals. In turn, the second feature can be explained by the spectral characteristics of radicals derived from dA [26], formed in the reaction of 30% of HO^•^ radicals. On the other hand, the optical spectrum recorded 1 μs after the pulse in an aqueous solution containing ct-DNA (Figure 5, right panel) is not very different from that recorded in a solution containing a mixture of four nucleosides, except for the fact that the absorption intensity is two-fold weaker. Interestingly, the time evolution of the radicals formed in both systems is different (clearly seen by comparison of the absorption spectra in the time domain between 50 μs and 1 ms) showing higher stability of radicals in ct-DNA.

#### 2.2.4. Single Stranded 12-Mer Oligodeoxynucleotides

The subsequent chemical systems subjected to irradiation were the aqueous solutions containing one of four single stranded 12-mer oligonucleotides (cf. Table 1): ODN1, ODN2, ODN3, or ODN4.

**Figure 6:**
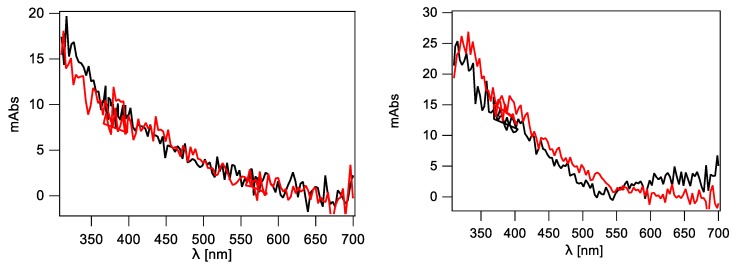
Transient absorption spectra recorded using ICCD in N_2_O-saturated phosphate buffered (50 mM) aqueous solutions containing 5′-CGT ATG GTA TCG-3′ (ODN1) (left panel) and 5′-CGA TGG GGT ACG-3′ (ODN3) (right panel) at natural pH: (**−**) 1 μs and (**−**) 50 μs after electron pulse.

Figure 6 shows the optical absorption spectra recorded 1 μs after the pulse for ODN1 and ODN3, which are very similar to the absorption spectrum recorded 1 μs after the pulse in aqueous solutions containing ct-DNA (Figure 5, right panel). Similar spectra are obtained for ODN2 and ODN4 (see Figure S1 in Supporting Information). Three of them (except ODN1) are characterized by the absorption band which can be assigned to the guanyl-type radical **3** (cf. Figure 3). Surprisingly, the spectrum recorded in aqueous solution of ODN1 (oligonucleotide containing four dG nucleotides) does not show optical features which can be assigned to this radical (Figure 6, left panel). It seems that the number of dG present in these single stranded 12-mer oligonucleotides is not the only factor determining the efficiency of the intermediate **3** formation. Perhaps, the peculiar conformation arrangement of ODN1 excludes the access of HO^•^ radical to the NH_2_ moiety for H-atom abstraction.

It is worth mentioning that the formation of the radical intermediate **3** and its tautomerization process (**3**→**4**) taking place on the ms scale is an important process, shown to occur in G-quadruplex through oxidation followed by deprotonation step [27,28,29].

#### 2.2.5. Double-Stranded 12-Mer Oligodeoxynucleotides

**Figure 7:**
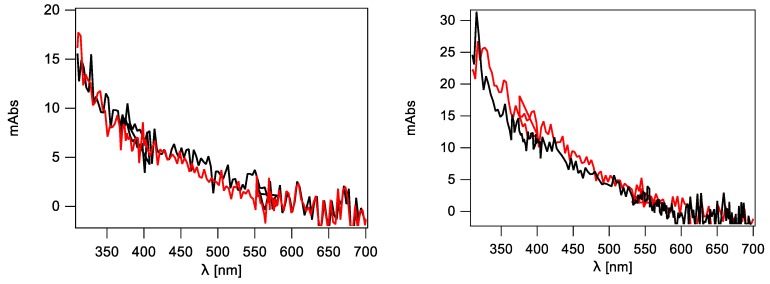
Transient absorption spectra recorded using ICCD in N_2_O-saturated phosphate buffered (50 mM) aqueous solutions containing (left panel) ds-(ODN1/ODN2) and (right panel) ds-(ODN3/ODN4) at natural pH: (**−**) 1 μs and (**−**) 50 μs after electron pulse.

The last two chemical systems subjected to irradiation were the aqueous solutions containing double-stranded (ds) 12-mer oligonucleotides: ODN1/ODN2 or ODN3/ODN4. In our previous studies, we used the same ds-oligonucleotide sequences for investigating the oxidation potential upon increasing the number of consecutive Gs [30]. The optical absorption spectra recorded 1 μs after the pulse in aqueous solutions containing one of two ds-oligonucleotides are very similar to each other (Figure 7) and resemble the spectrum recorded 1 μs after the pulse in aqueous solutions containing ct-DNA (Figure 5, right panel). Moreover, the time evolution of spectra recorded at 1 μs and 50 μs shows clearly that the formed radicals are stable within this time domain. The lack of the absorption band >600 nm indicates absence of guanyl-type radicals which might result from the specific structure of the double-stranded DNA (Figure 7). It is reasonable to assume that the HO^•^ radical is not able to reach the NH_2_ moiety for H-atom abstraction due to the steric encumbrance in ds-ODNs or the tautomerization process (**3**→**4**) is very fast with respect to our time-scale experiments.

### 2.3. γ-Radiolysis

#### 2.3.1. Hydroxyl Radical–Induced Formation of Purine Lesions in ct-DNA

The reactions of HO^•^ radicals with DNA were carried out using ct-DNA. Two preparation procedures of ct-DNA solutions for irradiation were used:

(i) the commercial ct-DNA solution containing 1 mM Tris-HCl, pH 7.5, with 1 mM NaCl and 1 mM EDTA was firstly lyophilized and then 200 µL of a N_2_O saturated ct-DNA aqueous solutions (0.5 mg/mL) at natural pH were prepared;
(ii) the commercial ct-DNA solution was desalted by ethanol precipitation (removal of the additives Tris-HCl, NaCl and EDTA) and then 200 µL of a N_2_O saturated ct-DNA (0.5 mg/mL) were prepared in 50 mM phosphate buffer, pH 7.2

All samples were irradiated (in triplicate) under steady-state conditions at different doses (0, 10, 20, 35, and 50 Gy). The quantification of the lesions was executed in two independent steps. Firstly, the sample was analyzed via an HPLC-UV system coupled with a sample collector. According to this first clean-up step, the quantification of the unmodified nucleosides took place, based on their absorbance at 260 nm, whereas, at their time-windows when the lesions are eluted, fractions were collected and pooled. The concentrated samples containing the modified DNA bases were injected subsequently to LC-MS/MS to be analyzed and quantified independently [10,16,31,32]. In the absence of the unmodified nucleosides, which cause solubility problems to arise, the sample can be concentrated prior to LC-MS/MS analysis increasing the overall sensitivity of the quantification method. The use of isotopically labeled lesions (Figure S2) maintains the reproducibility and the recovery of the quantification protocol within the levels that are generally accepted for reliability. The calibration curves for the quantification of the lesions and the list of MRM transitions employed for the quantification are reported in Figure S3 and Table S1, respectively.

**Figure 8:**
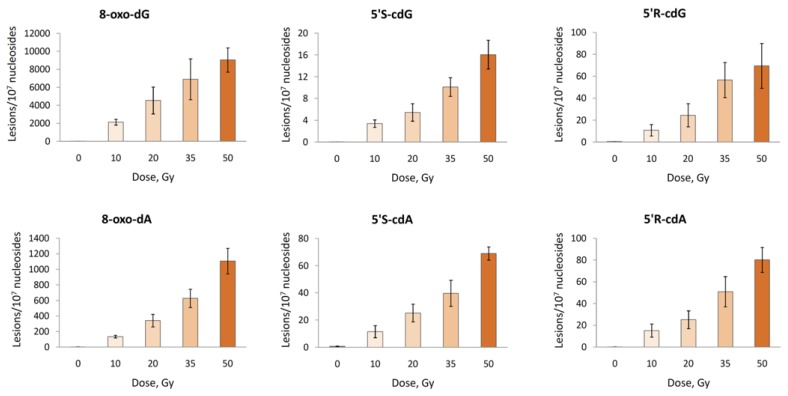
Radiation induced formation of 8-oxo-dG, 5′*S*-cdG, 5′*R*-cdG, 8-oxo-dA, 5′*S*-cdA and 5′*R*-cdA in ct-DNA Procedure (i). Each sample was exposed to 0, 10, 20, 35, and 50 Gy dose. The values represent the mean ± SD of *n* = 3 independent experiments.

The radiation induced formation of 8-oxo-dG, 5′*S*-cdG, 5′*R*-cdG, 8-oxo-dA, 5′*S*-cdA and 5′*R*-cdA in ct-DNA applying Procedure (i) is shown in Figure 8 (for data, see Table S2). As expected, the number of the lesions studied increases with the increment of the dose. The reaction product profile obtained employing Procedure (ii) (see Figure S4 and Table S3), where ct-DNA is treated for removing additives such as Tris-HCl, NaCl and EDTA, is comparable with the results gathered with Procedure (i); this indicates that the additives originally present in the commercial ct-DNA sample have no significant interference in the reaction outcome.

**Figure 9:**
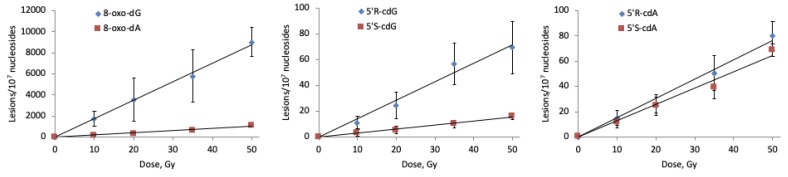
Radiation induced formation of 8-oxo-dG, 8-oxo-dA, 5′*R*-cdG, 5′*S*-cdG, 5′*R*-cdA, and 5′*S*-cdA in ct-DNA Procedure (i). Each sample was exposed to 0, 10, 20, 35, and 50 Gy dose in N_2_O-saturated aqueous solutions; the values represent the mean ± SD of *n* = 3 independent experiments.

From Figure 8, it becomes evident that the main reaction products detected are 8-oxo-dG and 8-oxo-dA, which are also formed in a ca. 10:1 ratio. The intramolecular cyclization products 5′*S*-cdG, 5′*R*-cdG, 5′*S*-cdA and 5′*R*-cdA are also formed, albeit in lower yields. We have further analyzed the data obtained employing Procedures (i) and (ii) (Tables S2 and S3) by plotting the number of each lesion formed vs. the radiation dose; the slope of the lines obtained (Figure 9 for Procedure (i) and Figure S5 for Procedure (ii)) represents the number of lesions formed per Gy which are reported in Table S3.

After having verified that Procedure (i) is indeed equivalent to (ii) (Table S4), the data obtained using both procedures at each dose were gathered and treated as a unique experiment, affording the data reported in column 2 of Table 2. The 5’*R* diastereomer is formed predominantly leading to 5′*R*/5′*S* ratio of 4.5 for cdG and 1.2 for cdA (column 3). It is very gratifying to observe how our data perfectly match previous results obtained and published by our group (columns 4 and 5) [33], considering different batches of ct-DNA, the labor-intensive enzymatic digestion/prepurification/enrichment of cPu lesions protocol, and the use of different analytical instrumentation. The level of total 8-oxo-Pu was found to be ~40-fold excess of total cPu lesions and the 8-oxo-dG/8-oxo-dA ratio of 7.7. The yields of four cPu lesions were found to be similar. It is worth recalling that 5′*R*/5′*S* ratios of 8.3 for cdG and 6 for cdA were obtained in water upon irradiation of free nucleosides [34,35], indicating that the diastereomer ratio is dependent on the molecular complexity. It is also worth mentioning that, in earlier work [36] on similar experiments, the level of lesions was reported to be much higher than in the present work for cdG and for cdA, (these data are reported in the last two columns of Table 2) and later the quantification protocol used was strongly criticized, being inappropriate for these measurements [16].

#### 2.3.2. Hydroxyl Radical–Induced Formation of Purine Lesions in Double Stranded 21-Mer Oligonucleotides

**Figure 10:**
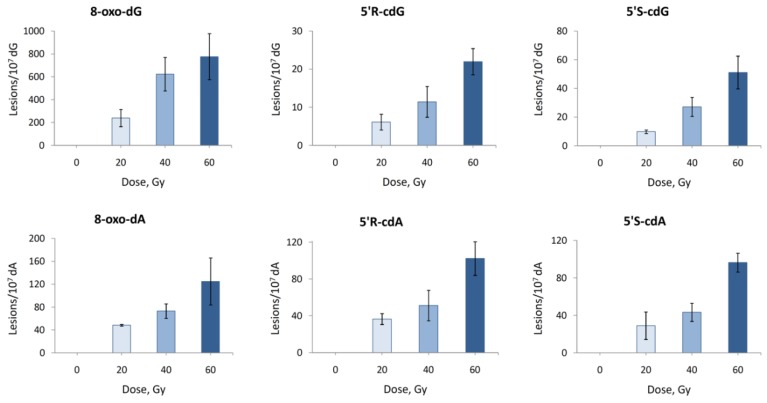
Radiation induced formation of 8-oxo-dG, 5′*S*-cdG, 5′*R*-cdG, 8-oxo-dA, 5′*S*-cdA and 5′*R*-cdA in double-stranded 21-mer oligonucleotides; Each sample was exposed to 0, 20, 40 and 60 Gy dose in N_2_O-saturated aqueous solutions. The values represent the mean ± SD of *n* = 3 independent experiments.

The reaction of HO^•^ radicals with double-stranded 21-mer oligonucleotide ODN5/ODN6 (see Table 1 for the ODNs sequences and Table S5, Figures S7–S9 for characterization) was studied under standard radiolytic conditions. For this purpose, 200 μL of N_2_O-saturated aqueous solutions containing ds-(ODN5/ODN6) (0.5 mg/mL) at natural pH were irradiated under steady-state conditions with a dose rate of 2.5 Gy min^−1^ at room temperature, followed by our optimized routine enzymatic oligonucleoside digestion and LC-MS/MS analysis. As expected, both 5′*R* and 5′*S* diastereomers of cdA and cdG as well as 8-oxo-dA and 8-oxo-dG were generated, and the number of lesions studied increased proportionally with the increment of the dose (Figure 10 and Table S6).

**Figure 11:**
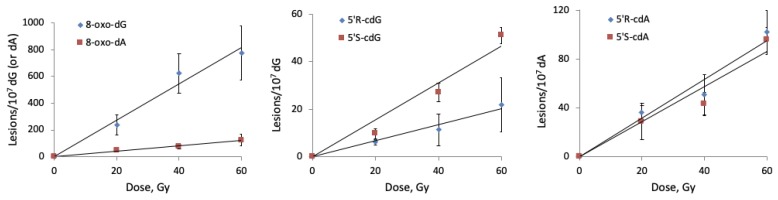
Radiation induced formation of 8-oxo-dG, 8-oxo-dA, 5′*R*-cdG, 5′*S*-cdG, 5′*R*-cdA, and 5′*S*-cdA in double-stranded 21-mer oligonucleotides; Each sample was exposed to 0, 20, 40 and 60 Gy dose in N_2_O-saturated aqueous solutions. The values represent the mean ± SD of *n* = 3 independent experiments.

From the analysis of the data reported in Figure 10, it turns out that the main reaction products detected are 8-oxo-dG and 8-oxo-dA, formed in an approximately 6.5:1 ratio. Intramolecular cyclization products 5′*S*-cdG, 5′*R*-cdG, 5′*S*-cdA and 5′*R*-cdA are also formed in the same fashion but in lower yields (Figure 10). Further analysis of the data reported in Figure 10 by plotting the number of each lesion detected vs. the radiation dose shows a linear correlation. The slope of the lines obtained (Figure 11) represents the number of lesions formed per Gy and these data are reported in Table 3. Analysis of the data shown in Table 3 proves that, in our experiments, the formation 8-oxo-dG is 6.4 times greater than 8-oxo-dA; regarding the cPu lesions detected, cdA lesions are higher than cdG and in 5′*R*/5′*S* ratios of ca 1.16 and 0.48, respectively. Although the diastereoisomeric ratio in cdA is similar to that observed in ct-DNA, in cdG, it is 10-fold smaller (0.48 vs. 4.5). It is also worth mentioning our previous work [37], where single-stranded and G-quadruplex of Tel22 d[AGGG(TTAGGG)_3_] and mutated Tel24 d[TTGGG(TTAGGG)_3_A] were exposed to HO^•^ radicals in similarity with ODN5 of ds-(ODN5/ODN6) in the present study. Indeed, for Tel22, it was reported 0.4 and 0.9 lesion/10^7^ dG/Gy for 5′*R*-cdG and 5′*S*-cdG, respectively, with a 5′*R*/5′*S* ratios of 0.44. All these data confirm that the diastereomer ratio is dependent on the molecular complexity and detailed theoretical calculations on the transition states are needed for a better understanding of C5′ radical cyclization in ds-ODNs.

### 2.4. Hydroxyl Radical Generated by Fenton Reactions and Formation of Purine Lesions in Double Stranded 21-Mer Oligonucleotides

Hydrogen peroxide (H_2_O_2_) reacts with the reduced-state transition metal ions, like Fe^2+^ or Cu^1+^, to give HO^•^ radicals (reaction 5) [38,39]:

H_2_O_2_ + Fe^2+^ (Cu^1+^) → HO^−^ + HO^•^ + Fe^3+^ (Cu^2+^). (5)

One-electron reduction of hydrogen peroxide occurs with a reduction potential of +0.38 V (H_2_O_2_,H^+^/H_2_O,HO^•^, pH 7, vs. NHE). Thus, the relatively long-lived oxidant H_2_O_2_ upon reduction generates a potent and indiscriminant oxidant, like HO^•^ radical.

This reaction, referred to as the Fenton reaction, has been reported to be responsible for some of the toxicity associated with H_2_O_2_ in vivo. The reduction of H_2_O_2_ in biological systems can occur via reaction with the reduced forms of several redox active metals such as the ferrous ion (Fe^2+^) or cuprous ion (Cu^1+^). H_2_O_2_ toxicity is highly dependent on the presence/location of reactive forms of Fe^2+^ or Cu^1+^ ions [40].

In this section, the role of HO^•^ radicals generated by Fenton reaction (5) with ds-oligodeoxynucleotides ds-(ODN5/ODN6) was investigated in some details. The measurements of cPu lesions in ct-DNA by Fenton-type reagents was reported by Wang and coworkers [41]. They used Cu(II) or Fe(II) 12.5 µM, H_2_O_2_ 100 µM and ascorbate 1 mM with proportional increments up to 1600 µM of H_2_O_2_ in 250 µL solution containing 75 µg of ct-DNA. For our studies, we used the same concentration ratio of Fenton-type reagents and followed an analogous approach.

**Figure 12:**
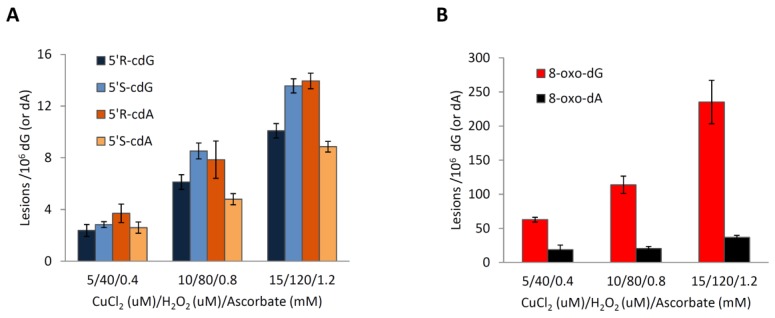
CuCl_2_/H_2_O_2_/Ascorbate-induced formation of (**A**) 5′*R*-cdG, 5′*S*-cdG, 5′*R*-cdA and 5′*S*-cdA and (**B**) 8-oxo-dG and 8-oxo-dA in ds-ODNs. The numbers represent the mean value (±standard deviation) of *n* = 3 independent experiments.

The reaction was explored employing three different systems that consisted of 50 µg of ds-(ODN5/ODN6) in 200 µL solution and proportional increment of CuCl_2_, H_2_O_2_ and ascorbate: 5 µM/40 µM/0.4 mM, 10 µM/80 µM/0.8 mM, and 15 µM/120 µM/1.2 mM, respectively (Table S7). Observing Figure 12 and Table S7, it becomes clear that, in all experiments, 5′*R*-cdG, 5′*S*-cdG, 5′*R*-cdA, 5′*S*-cdA, 8-oxo-dG, and 8-oxo-dA lesions are formed. It is also gratifying to observe an increment in the lesions number with the increased concentration of the reagents employed in the Fenton system (Figure 12 and Table S8). The main lesion detected in the three systems studied is 8-oxo-dG followed by 8-oxo-dA. It is interesting to point out that the ratio between 8-oxo-dG and 8-oxo-dA increases as the concentration of the reagents employed increases; that is, 8-oxo-dG/8-oxo-dA ratio 3.4:1, 5.6:1, 6.4:1 for CuCl_2_/H_2_O_2_/ascorbate: 5 µM/40 µM/0.4 mM, 10 µM/80 µM/0.8 mM, and 15 µM/120 µM/1.2 mM, respectively (Figure 12B). This result is in agreement with the fact that the oxidation of dG proceeds faster than the oxidation of dA [42]. Regarding the formation of the 5′,8-cyclopurines, the four assessed lesions (5′*R*-cdG, 5′*S*-cdG, 5′*R*-CdA and 5′*S*-cdA) increase with the increment of the reagents concentration employed in the Fenton reaction (Figure 12A); it should be mentioned that the 5′*R*/5′*S* ratio for both the cdG and the cdA lesions remains almost constant in the three reaction conditions studied being ~0.76 for cdG and ~1.56 for cdA (Table S9).

**Figure 13:**
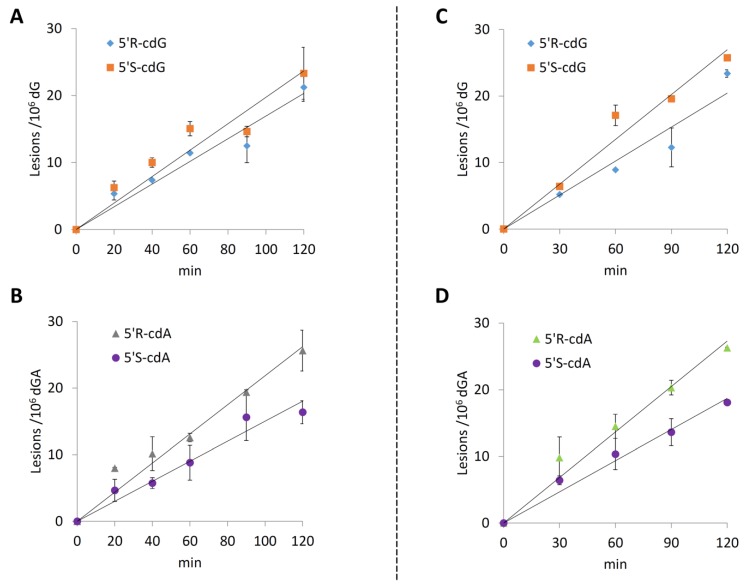
Kinetic study by Fenton reaction. (A and B): CuCl_2_/H_2_O_2_/Ascorbate-induced formation of 5′*R*-cdG, 5′*S*-cdG, 5′*R*-cdA and 5′*S*-cdA at 0, 20, 40, 60, 90 and 120 min. ds-(ODN5/ODN6) treated with CuCl_2_ (15 µM), H_2_O_2_ (120 µM), Ascorbate (1.2 mM); (C and D): Fe^2+^/H_2_O_2_/Ascorbate-induced formation of 5′*R*-cdG, 5′*S*-cdG, 5′*R*-cdA and 5′*S*-cdA at 0, 30, 60, 90 and 120 min. ds-(ODN5/ODN6) treated with Fe^2+^ (15 µM), H_2_O_2_ (120 µM), Ascorbate (1.2 mM). All points are the mean value (± standard deviation) of *n* = 2 independent experiments.

**Figure 14:**
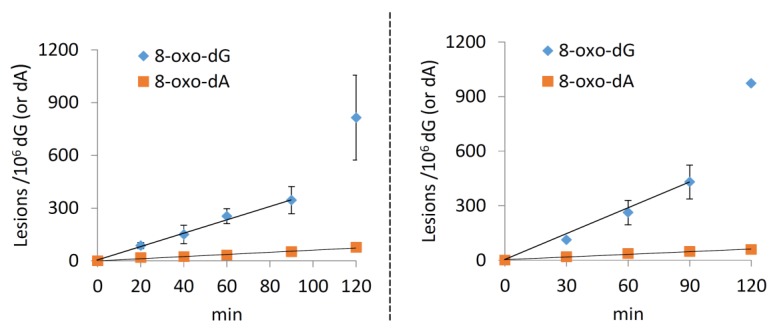
Kinetic study by Fenton reaction. (Left side): CuCl2/H2O2/Ascorbate-induced formation of 8-oxo-dG and 8-oxo-dA at 0, 20, 40, 60, 90 and 120 min. ds-(ODN5/ODN6) treated with CuCl_2_ (15 µM), H_2_O_2_ (120 µM); (Right side): Fe^2+^/H_2_O_2_/Ascorbate-induced formation of 8-oxo-dG and 8-oxo-dA at 0, 30, 60, 90 and 120 min. ds-(ODN5/ODN6) treated with Fe^2+^ (15 µM), H_2_O_2_ (120 µM), Ascorbate (1.2 mM). All points are the mean value (± standard deviation) of *n* = 2 independent experiments.

After analyzing the reaction outcome at various proportional increments of CuCl_2_, H_2_O_2_, and ascorbate, it was deemed proper to study the reaction evolution with time. For doing so, the most reactive reaction conditions previously studied were employed, that is, CuCl_2_, H_2_O_2_ and ascorbate 15 µM/120 µM/1.2 mM, respectively. As expected, in all the experiments, an increment of the reaction products was observed with the increase of the reaction time (Tables S10 and S11). Figure 13 (A and B) shows the plots of the mean value of each purine lesion studied at different reaction times; analogously, Figure 14 (left side) shows the plots for 8-oxo-dA and 8-oxo-dG. The linear ds-ODNs lesion rates could just reflect that the reaction half-life is much longer than the experimental time-frame, *t*_1/2_ >> 2 h. The slope of the lines can be interpreted as the number of lesions produced in 1 min of reaction (Table 4). The data reported in Table 4 (column 2) show that the main lesions detected are 8-oxo-dG (3.88 lesions per minute) and 8-oxo-dA (0.60 lesions per minute) in a 6.5:1 ratio, respectively. The 5′*R*-cdG, 5′*S*-cdG, 5′*R*-cdA and 5′*S*-cdA lesions are also formed, albeit in lower yields ranging between 0.14 and 0.20 lesions per minute.

We continued our investigations on ds-(ODN5/ODN6) lesion formation by replacing Cu^+^ with Fe^2+^ in the Fenton system, using a preestablished concentration of Fe(II)/H_2_O_2_/ascorbate: 15 µM/120 µM/1.2, which corresponds to the most efficient ratio of reagents towards ds-ODNs lesion formation when using Cu^+^ as the metal source. As expected, the Fenton system employing iron also induced a time-dependent formation of the assessed purine lesions (Tables S13 and S14). Figure 13 (C and D) shows the plots of the mean value of each purine lesion studied at different reaction times; analogously, Figure 14 (left side) shows the plots for 8-oxo-dA and 8-oxo-dG. The data reported in Table 4 (column 3) show the number of lesions produced in 1 min, showing that by replacing Cu^+^ with Fe^2+^ in these Fenton-type of reactions the outcome is similar. It is also gratifying to see that the 5′*R*/5′*S* ratio in the two Fenton systems are similar, i.e., after 120 min for the cuprous ion the ratios are 0.91 for cdG and 1.57 for cdA, whereas, for the ferrous ion, the ratios are 0.91 for cdG and 1.45 for cdA (cf. Table S12 with Table S15).

**Figure 15:**
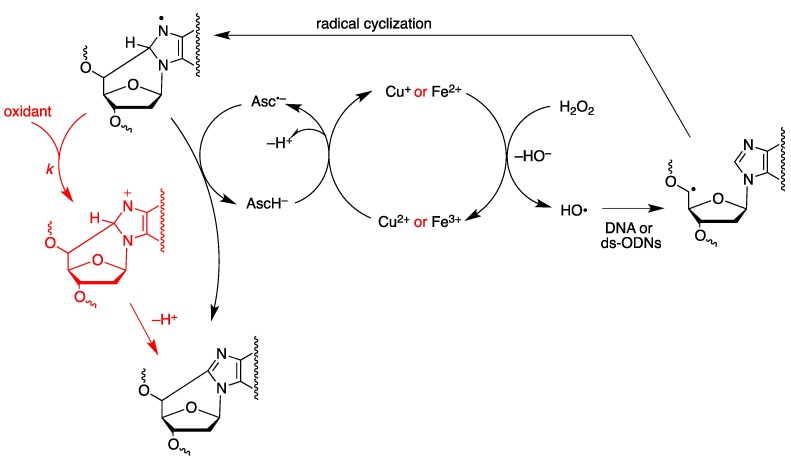
The conceived reaction mechanism for the formation of cPu lesion in ds-ODNs by Fenton-type reactions.

Figure 15 summarizes the reaction mechanism we conceived for the above described Fenton-type reactions. The Fenton reaction produces HO^•^ radical and oxidation of copper and iron (Cu^2+^ and Fe^3+^). The role of ascorbate is to maintain copper and iron in the reduced state (Cu^+^ and Fe^2+^) with the formation of ascorbyl radical anion (Asc^•−^). There is a plethora of paths for the reaction of HO^•^ radical with DNA or ds-ODNs [1,2]. It can be estimated that 2–3% only occur by hydrogen abstraction at C5’ position in the purine nucleotide moieties leading to C5′ radical. After the intramolecular addition to C8–N7 double bond, the formation of an heteroaromatic aminyl radical results. This radical can be easily oxidized by Cu^2+^ or Fe^3+^ to afford the final lesion after deprotonation. At the nucleoside level, we reported a rate constant of 8.3 × 10^8^ M^−1^s^−1^ for the aminyl radical with Fe(CN)_6_^3−^ [43,44]. Alternatively, if the [Asc^•−^] is built-up in a fairly high steady-state concentration, there will be the possibility of radical disproportionation with the regeneration of ascorbate and the cPu lesion formation within the biomacromolecule. It is worth mentioning that, after 60 min, the reactions were quenched by adding 0.4 mg L-methionine as described in the protocol of Wang [41], more likely in order to eliminate remaining oxidizing species. However, control experiments without L-methionine gave similar results (results not shown).

## 3. Materials and Methods

### 3.1. Chemicals, Reagents and Enzymes

Nuclease P1 from Penicillium citrinum, phosphodieasterase I and II, alkaline phosphatase from bovine intestinal mucosa, DNase I and DNase II, benzonase 99%, BHT, deferoxamine mesylate and pentostatin were purchased from Sigma-Aldrich (Steinheim, Germany). RNase T1 was from Thermo Fisher Scientific (Waltham, MA, USA) and RNase A from Roche Diagnostic GmbH, (Mannheim, Germany). The 3 kDa cut-off filters were obtained from Millipore (Bedford, OH, USA). Chemicals for the synthesis of oligonucleotides were purchased from Sigma Aldrich, Fluka and Link Technologies. CuCl_2_, L-methionine, L-ascorbic acid and alkaline phosphatase were purchased from Sigma-Aldrich. Hydrogen peroxide (30%) and solvents (HPLC-grade) were purchased from Fisher Scientific. 2′-deoxyadenosine monohydrate and 2′-deoxyaguanosine were purchased from Berry & Associates Inc. (Dexter, USA). Isotopic labeled internal standards of 5′*R*-cdA, 5′*S*-cdA, 5′*R*-cdG, 5′*S*-cdG, 8-oxo-dG and 8-oxo-dA (see Supporting Information) were prepared according to the previously reported procedures [31]. Ultrapure water (18.3 MΩcm) distilled and deionized water (Milli-Q water) were purified by a Milli-Q system (Merck-Millipore, Bedford, OH, USA).

### 3.2. Oligodeoxynucleotides (ODNs) Synthesis and Purification

ODNs were prepared by automated synthesis using the DMT- and β-(cyanoethyl)-phosphoramidite method, on CPG supports (500 Å), with an Expedite 8900 DNA synthesizer (Applied Biosystems, Foster City, CA, USA) at 1 µmol scale. Following their synthesis, the DMTr-on ODNs were cleaved from the solid support and deprotected by the method of two syringes using an AMA reagent (NH_4_OH (30%)/CH_3_NH_2_ (40%) 1:1) for 10 min at room temperature. The AMA solution containing the cleaved ODN was placed in a sealed vial and heated for 15 min at 55 °C. The solvent was then removed in a Speedvac. The crude 5′-DMT-on oligomers were purified and detritylated on-column by RP-HPLC (Grace Vydac C18 column, 5 µm, 50 × 22 mm). The ODNs were further purified by SAX HPLC (preparative DNA Pac PA-100 column, 5 µm, 22 × 250 mm). TRIS.HCl 25 mM, pH = 8 (buffer A) and TRIS. HCl 25 mM, NaClO_4_ 0.5 M, pH 8.0 (buffer B) were used at a flow rate of 9 mL/min eluting with 2–30% B in 30 min, 30% B for 10 min, then 30–45% B in 5 min monitoring at 254 nm. The purified fractions were concentrated, desalted on Water SepPakTM-C18-cartridges (Milford, MA, USA) and lyophilized again. The final DNA yield was estimated by UV absorption in aqueous solution measured at 254 nm on a Cary 100 UV/Vis Spectrometer (Agilent, Cernusco sul Naviglio, Italy) following standard procedures. Electrospray Ionization (ESI) was used to characterize the purified ODNs. Maldi-TOF mass spectrometry (ODN1 to ODN4) [30] and Electrospray Ionization (ESI) (ODN5 and ODN6) were used to characterize the purified ODNs (see Table S5 and Figure S7).

### 3.3. Preparation of Double Stranded Oligonucleotide Substrates

The oligonucleotide strands were annealed to the complementary strands in equimolar concentrations in buffer solution containing 10 mM sodium phosphate, 100 mM NaCl, 0.1 mM EDTA, pH 7.2. The substrates were constructed by heating the two strands of the substrates at 90 °C for 10 min and subsequently allowing the temperature to slowly drop down to the room temperature (25 °C). Melting temperatures (Tm) of the substrates were measured with a Cary 100 UV/Vis spectrometer (Agilent, Cernusco sul Naviglio, Italy) using a 1 mL quartz cuvette with a 1 cm path length. This allowed monitoring of the absorbance of the solutions at 260 nm as a function of the temperature. The temperature cycles were recorded from 20 to 80 °C per strand with a temperature controller at a heating rate of 0.3 °C/min. UV melting curves of 21-mer duplexes ODN5/ODN6 (See Figure S8).

CD spectra were recorded on a Jasco *J-710* spectropolarimeter (Cremella, Italy) using a quartz cuvette (0.1 cm optical path length) at a scanning speed of 50 nm/min with 1 s response time. Measurements at the range of 200–360 nm were the average of four accumulations at 295 K and smoothed with Origin, Version 8.00 program (OriginLab Corporation, Northampton, MA, USA). The 21-mer duplexes ODN5/ODN6 contained an aqueous solution of 50 mM sodium phosphate, pH 7.2 and 50 μM double stranded oligonucleotide substrates (Figure S9. The reported spectrum was obtained by subtracting the spectrum of blank (aqueous solution of sodium phosphate buffer).

### 3.4. Pulse Radiolysis

Pulse radiolysis experiments with time-resolved UV-vis optical absorption detection were carried out at the Institute of Nuclear Chemistry and Technology in Warsaw, Poland. The linear electron accelerator (LAE 10) delivering 10 ns pulses with electron energy about 10 MeV was applied as a source of irradiation. The 150 W xenon arc lamp E7536 (Hamamatsu, Shizuoka, Japan) was used as a monitoring light source. The respective wavelengths were selected by MSH 301 (Lot Oriel Gruppe) motorized monochromator/spectrograph with two optical output ports. The time dependent intensity of the analyzing light was measured by means of photomultiplier (PMT) R955 (Hamamatsu, Shizuoka, Japan). A signal from detector was digitized using a WaveSurfer 104MXs-B (1 GHz, 10 GS/s, LeCroy) oscilloscope. Alternatively, iSTAR Intensified Charge-Coupled Device (ICCD) (A-DH720-18F-03) detector with W-type photocathode and 18 mm Multi-Channel Plate (MCP) image intensifier was used for transient spectra measurements. Minimum optical gate width of this detector was <5 ns with a spectral range of 180–850 nm. In order to avoid photodecomposition and/or photobleaching effects in the samples, the UV or VIS cut-off filters were used. However, no evidence of such effects was found within the time domains monitored. Water filter was used to eliminate near IR wavelengths. Optical path of microcells was 1 cm with a total volume of irradiated solution about 300 μl. All experiments were carried out at the ambient temperature ~22 °C. The spectral range which can be covered with the existing pulse radiolysis set-up is comprised between 300–700 nm.

The total dose per electron pulse was determined before each series of experiments by a thiocyanate dosimeter (N_2_O-saturated aqueous solution containing 10 mM KSCN) using *G* × ε = 5.048 × 10^−3^ mol J^−1^ M^−1^ cm^−1^ for the (SCN)_2_^•−^ radical anion at λ = 472 nm.

### 3.5. γ-Radiolysis Experiments

Each sample of ds-DNA (50 µg) dissolved in 200 µL of phosphate buffer 50 mM was placed in a 2 mL glass vial. Irradiations were performed at room temperature (22 ± 2 °C) using a ^60^Co-Gammacell at different doses (dose rates: 2.5 Gy/min). The exact absorbed radiation dose was determined with the Fricke chemical dosimeter, by taking G(Fe^3+^) 1.61 μmol J^−1^. In particular, the irradiation doses used were 20, 40, and 60 Gy, and the solutions were saturated by N_2_O. All the irradiation experiments were performed in triplicates.

### 3.6. Fenton-Type Reagent Treatments of ds-ODNs

#### 3.6.1. CuCl_2_ with L-Methionine

Aliquots of ds-ODNs (50 μg) were incubated with CuCl_2_ (5–15 μM), H_2_O_2_ (40–120 μM), Ascorbate (0.40–1.2 mM) in a 200 μL solution containing 25 mM NaCl and 50 mM phosphate (pH 7.2) at room temperature under aerobic conditions for 60 min. Ascorbate was added to maintain copper in the reduced state (Cu^+^), so that it could participate in the Fenton reaction. Chemicals used in the Fenton-type reagent treatment of ds-ODNs were freshly prepared in doubly distilled water. After 60 min, the reactions were terminated by adding 0.4 mg L-methionine (control treatments were performed also without L-methionine), and the ODN samples were desalted by ethanol precipitation (10% volume of 3 M sodium acetate, pH 5.2 and 3 volumes of 100% Ethanol). The samples were mixed and frozen overnight at −20 °C. In the morning, the samples were centrifuged at 13,000 RPM at 4 degrees for 45 min. The supernatant was decanted. The pellet was washed and centrifuged again, for only 15 min, with 80% EtOH. The supernatant was decanted and the pellet air dried.

#### 3.6.2. Kinetic Study by Cu^2+^/H_2_O_2_ of ds-ODNs with L-Methionine

Aliquots of ds-ODNs (16 μg) were incubated with CuCl_2_ (15 μM), H_2_O_2_ (120 μM), Ascorbate (1.2 mM) in a 64 μL solution containing 25 mM NaCl and 50 mM phosphate (pH 7.2) at room temperature under aerobic conditions. After 20–40–60–90–120 min, the reactions were terminated by adding 115 µg of L-methionine, and the DNA samples were desalted by ethanol precipitation (10% volume of 3M sodium acetate, pH 5.2 and 3 volumes of 100% Ethanol). The samples were mixed and frozen overnight at −20 °C. In the morning, the samples were centrifuged at 13,000 RPM at 4 degrees for 45 min. The supernatant was decanted. The pellet was washed and centrifuged again, for only 15 min, with 80% EtOH. The supernatant was decanted and the pellet air dried.

#### 3.6.3. Kinetic Study by Fe^2+^/H_2_O_2_) of ds-ODNs with L-Methionine

Aliquots of ds-ODNs (50 μg) were incubated with Fe(NH_4_)_2_(SO_4_)_2_^•^6H_2_O (15 μM), H_2_O_2_ (120 μM), Ascorbate (1.2 mM) in a 200 μL solution containing 25 mM NaCl and 50 mM phosphate (pH 7.2) at room temperature under aerobic conditions. After 30–60–90–120 min, the reactions were terminated by adding 336 µg of L-methionine, and the DNA samples were desalted by ethanol precipitation (10% volume of 3 M sodium acetate, pH 5.2 and 3 volumes of 100% Ethanol). The samples were mixed and freeze overnight at –20 °C. In the morning, the samples were centrifuged at 13,000 RPM at 4 degrees for 45 min. The supernatant was decanted. The pellet was washed and centrifuged again, for only 15 min, with 80% EtOH. The supernatant was decanted and the pellet air dried. Fenton-type reagent treatment (Fe^2+^/H_2_O_2_) of ds-ODNs was performed also without L-methionine to elucidate the role of L-methionine in the progress of the reaction.

### 3.7. Enzymatic Digestion of the ct-DNA and ds-ODNs

In addition, 50 μg of ct-DNA or ds-ODN were dissolved in 100 μL of Ar flushed 10 mM Tris-HCl (pH 7.9), containing 10 mM MgCl_2_, 50 mM NaCl, 0.2 mM pentostatin, 5 μM BHT and 3 mM deferoxamine and the internal standards were added ([^15^N_5_]-5′*S*-cdA, [^15^N_5_]-5′*R*-cdA, [^15^N_5_]-5′*S*-cdG, [^15^N_5_]-5′R-cdG, [^15^N_5_]-8-oxo-dG and [^15^N_5_]-8-oxo-dA) as previously described [31]. Furthermore, 3 U of benzonase (in 20 mM Tris-HCl pH 8.0, 2 mM MgCl_2_ and 20 mM NaCl), 4 mU phosphodiesterase I, 3 U DNAse I, 2 mU of phosphodiesterase II and 2 U of alkaline phosphatase were added and the mixture was incubated at 37 °C. After 21 h, 35 μL of Ar flushed buffer containing 0.3 M AcONa (pH 5.6) and 10 mM ZnCl_2_ were added along with 0.5 U of Nuclease P1 (in 30 mM AcONa pH 5.3, 5 mM ZnCl_2_ and 50 mM NaCl), 4 mU PDE II and 125 mU of DNAse II and the mixture was further incubated at 37°C for extra 21 h. A step-quenching with 1% formic acid solution (final pH ~ 7) was followed, the digestion mixture was placed in a microspin filter (3 kDa) and the enzymes were filtered off by centrifugation at 14,000× *g* (4 °C) for 20 min. Subsequently, the filtrate was freeze-dried before HPLC analysis, clean-up, and enrichment.

### 3.8. HPLC Analysis and Quantification of Modified Nucleosides by Stable Isotope LC-MS/MS

The quantification of the modified nucleosides (in lesions/10^6^ nucleosides units) in the enzymatically digested samples (spiked with the ^15^N-labeled nucleosides) was based on the parallel quantification of the unmodified nucleosides after HPLC clean-up and sample enrichment and the quantification of the single lesions by stable isotope dilution LC-MS/MS analysis [16]. HPLC-UV clean-up and enrichment of the enzyme free samples were performed using a gradient program (2 mM ammonium formate, acetonitrile and methanol) while the fractions containing the lesions were collected, freeze-dried, pooled, freeze-dried again, and redissolved in Milli-Q water before been injected for LC-MS/MS analysis. Detection was performed in multiple reaction monitoring mode (MRM) using the two most intense and characteristic precursor/product ion transitions for each DNA lesion [32,33].

## 4. Conclusions

Pulse radiolysis in a series of 12 mer as ss-ODNs or ds-ODNs and ct-DNA gives only limited information. In particular, (i) the time evolution of spectra recorded at 1 μs and 50 μs shows that the formed radicals are stable within this time domain, and, (ii) in three ss-ODNs cases, it shows the absorption band > 600 nm that can be assigned to guanyl-type radicals and its decay due to the tautomerization.

The use of cPu lesions as a candidate marker of DNA damage is increasingly appreciated [4]. They offer, together with 8-oxo-Pu lesions, a profile of purine lesions with different properties: cPu as markers of HO^•^ radical damage in aging and diseases (repaired by NER), whereas 8-oxo-Pu are the results of various oxidizing species including HO^•^ radical (repaired by BER). In the present work, the simultaneous measurement of the four cPu and two 8-oxo-Pu upon reaction of genetic material with HO^•^ radicals, generated either by γ-radiolysis or Fenton-type reaction, contribute to greater knowledge on an absolute level of lesions according to the method of HO^•^ radical generation, relative abundance between cPu and 8-oxo-Pu, and the diastereomer ratio in cPu, with the latter one being associated with molecular complexity. The robustness of analytical protocol, which does not produce artifactual oxidations, was also provided by dose curve dependence and comparison of different methods of HO^•^ radical generation, thus rendering our results useful to shed light on disagreements in the literature regarding the formation of DNA purine lesions [16].

## Figures and Tables

**Table 1:** The sequences of the single stranded (ss) oligodeoxynucleotides (ODN) used in this study.

Strands	Sequence (5′-3′)	Length
ODN1	CGT ATG GTA TCG	12
ODN2	CGA TAC CAT ACG	12
ODN3	CGA TGG GGT ACG	12
ODN4	CGT ACC CCA TCG	12
ODN5	GGG (TTA GGG)_3_	21
ODN6	CCC (TAA CCC)_3_	21

**Table 2:** The levels (lesions/10^7^ nu/Gy) of 8-oxo-dG, 8-oxo-dA, 5′*R*-cdG, 5′*S*-cdG, 5′*R*-cdA and 5′*S*-cdA from the irradiation of N_2_O saturated ct-DNA (0.5 mg/mL) aqueous solutions.

Lesion	Lesions/10^7^ nu/Gy *^a^*	5′*R*/5′*S ^a^*	Lesions/10^7^ nu/Gy *^b^*	5′*R*/5′*S* ^*b*^	Lesions/10^7^ nu/Gy *^c^*	5′*R*/5′*S* ^*c*^
8-oxo-dG	171.8 ± 13.0		200.1 ± 3.03		780	
8-oxo-dA	22.22 ± 1.25		28.04 ± 0.46		72	
5′R-cdG	1.40 ± 0.12	4.5	2.98 ± 0.10	4.7	151 *^d^*	~3
5′S-cdG	0.31 ± 0.02		0.64 ± 0.06		50 *^d^*	
5′R-cdA	1.55 ± 0.08	1.2	1.47 ± 0.14	1.5	114^*d*^	~4
5′S-cdA	1.30 ± 0.06		0.95 ± 0.07		28^*d*^	

*^a^* This work; Procedure (i)+(ii); *^b^* From Ref. [33]; The original data are plotted in Supporting information (Figure S6). In the Ref. [33] only cdG and cdA together with 5′*R*/5′*S* ratio were reported and the diastereoisomeric ratio of cdG was erroneously reported to be 7 instead of 4.7; *^c^* From Ref. [36], where only cdG and cdA together with 5′*R*/5′*S* ratios were given; *^d^* The values of each diastereoisomer were calculated from the cdG (201 lesions/10^7^nu/Gy) or cdA (142 lesions/10^7^nu/Gy) taking into consideration the 5′*R*/5′*S* ratio [36].

**Table 3:** The levels (lesions/10^7^ nu/Gy) of 8-oxo-dG, 8-oxo-dA, 5′R-cdA, 5′S-cdA, 5′*R*-cdG and 5′*S*-cdG in irradiated ds-ODNs.

	ds-(ODN5/ODN6)	
Lesion	Lesions/10^7^ dG/Gy	Lesions/10^7^ dA/Gy
8-oxo-dG	13.49 ± 1.87	
8-oxo-dA		2.11 ± 0.30
5′*R*-cdG	0.32 ± 0.04	
5′*S*-cdG	0.67 ± 0.18	
5′*R*-cdA		1.60 ± 0.29
5′*S*-cdA		1.38 ± 0.27

**Table 4:** The line slope for each lesion obtained from the plot of the mean value of each purine lesion studied at different reaction times as reported in Figure 13 for cPu and Figure 14 for 8-oxo-Pu.

Lesions	From Cu^1+^	From Fe^2+^
5′*R*-cdG	0.16	0.18
5′*S*-cdG	0.17	0.21
5′*R*-cdA	0.20	0.21
5′*S*-cdA	0.14	0.14
8-oxo-dG	3.88	4.81
8-oxo-dA	0.60	0.49

## Supplementary Materials

The following are available online at https://www.mdpi.com/1420-3049/24/21/3860/s1, Figure S1: Transient absorption spectra, Figure S2: ^15^*N* isotopic labeled compounds, Figure S3: Calibration curves for the quantification of the lesions, Figure S4 and S5: Radiation induced formation of cPu and 8-oxo-Pu in ct-DNA Procedure (ii), Figure S6: Radiation induced formation of cPu and 8-oxo-Pu in ds-ODNs, Figure S7: ESI spectra of ODN5 and ODN6, Figure S8: UV melting curves of 21-mer duplexes, Figure S9: CD spectra of 21-mer duplexes, Table S1: A list of MRM transitions, Table S2: Total amount of cPu and 8-oxo-Pu in DNA applying Procedure (i), Table S3: Total amount of cPu and 8-oxo-Pu in DNA applying Procedure (ii), Table S4: The levels cPu and 8-oxo-Pu in ct-DNA DNA, Table S5: Sequences and molecular masses of the synthesized ODNs, Table S6: Total amount of cPu and 8-oxo-Pu in double-stranded 21-mer oligonucleotides, Table S7: The levels of cPu and 8-oxo-Pu upon Fenton-type reagent treatment of ds-ODNs, Table S8: Total amount of 8-oxo-purine lesions and cPu lesions upon Fenton-type reagent treatment of ds-ODNs, Table S9. The diastereoisomeric ratios (5′*R*/5′*S*) for both cdG and cdA upon Fenton-type reagent treatment of ds-ODNs, Table S10: The levels of cPu and 8-oxo-Pu upon treatment of ds-ODNs with Fenton-type reagents (copper), Table S11: Total amount of 8-oxo-purine lesions and cPu lesions formation upon treatment of ds-ODNs with Fenton-type reagents (copper), Table S12: The diastereoisomeric ratios (5′*R*/5′*S*) for both cdG and cdA upon treatment of ds-ODNs with Fenton-type reagents (copper), Table S13: The levels of cPu and 8-oxo-Pu upon treatment of ds-ODNs with Fenton-type reagents (iron), Table S14. Total amount of 8-oxo-purine lesions and cPu lesions formation upon treatment of ds-ODNs with Fenton-type reagents (iron), Table S15: The diastereoisomeric ratios (5′*R*/5′*S*) for both cdG and cdA upon treatment of ds-ODNs with Fenton-type reagents (iron).
