# Supplementary material for: New Insights into the Reaction Paths of Hydroxyl Radicals with Purine Moieties in DNA and Double-Stranded Oligodeoxynucleotides
Source: Molecules. 2019 Oct 26;24(21):3860. doi: 10.3390/molecules24213860 (PMC6865195; doi:10.3390/molecules24213860)
Supplement: Supplementary file 1 [file molecules-24-03860-s001.pdf]

molecules

Supplementary Material

New insights into the reaction paths of hydroxyl radicals with purine moieties in DNA and double-stranded oligonucleotides

Chryssostomos Chatgilialoglu*, Marios G. Krokidis, Annalisa Masi, Sebastian Barata-Vallejo, Carla Ferreri, Michael A. Terzidis, Tomasz Szreder and Krzysztof Bobrowski

* Correspondence: chrys@isof.cnr.it; Tel.: +39-051-639-8309

Table of Contents

Figure S1 and Figure S2	page 2
Figure S3	page 3
Table S1 and Table S2	page 4
Table S3 and Figure S4	Page 5
Figure S5 and Table S4	Page 6
Figure S6	Page 7
Table S5 and Figure S7	Page 8
Figure S8 and Figure S9	page 9
Table S6 and Table S7	Page 10
Table S8 and Table S9	Page 11
Table S10 and Table S11	Page 12
Table S12 and Table S13	Page 13
Table S14 and Table S15	Page 14

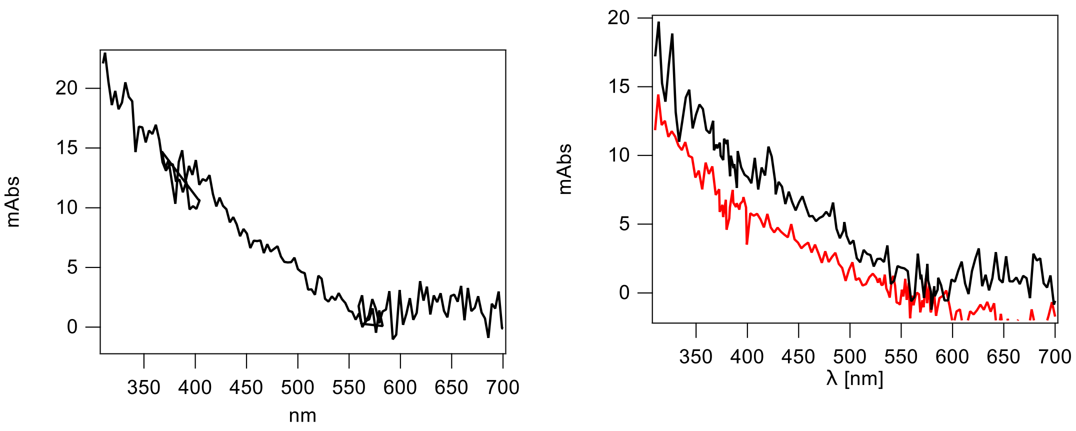

Figure S1. Transient absorption spectra recorded using ICCD in N_2O-saturated phosphate buffered (50 mM) aqueous solutions containing (left panel) 5'-CGA TAC CAT ACG-3' (ODN2) and (right panel) 5'-CGT ACC CCA TCG-3'(ODN4) at natural pH: (–) 1 µs and (–) 50 µs after electron pulse.

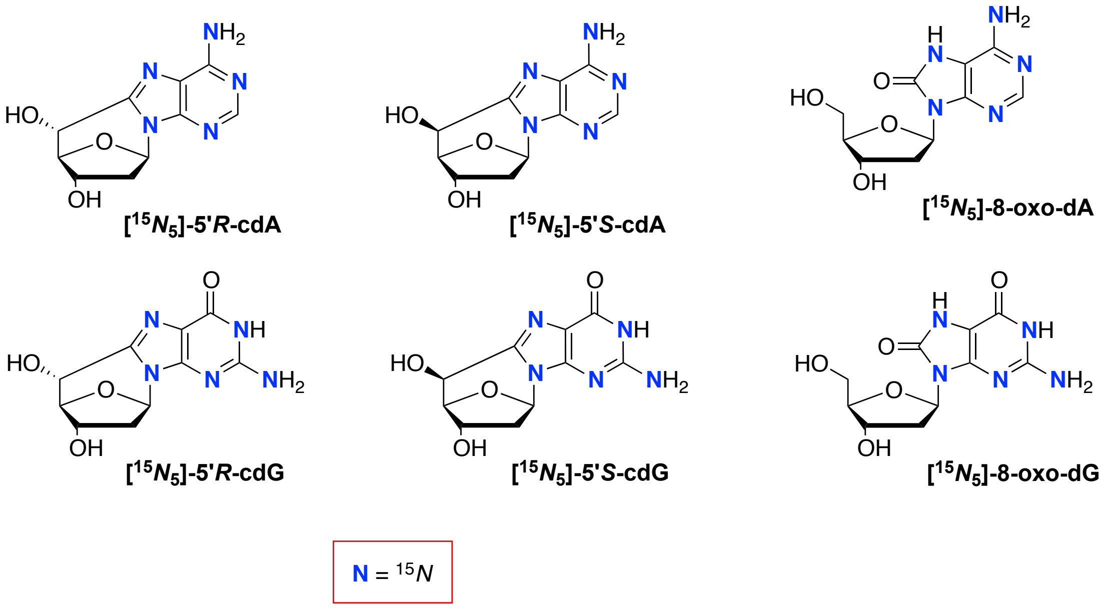

Figure S2. ^{15}N isotopic labeled compounds.

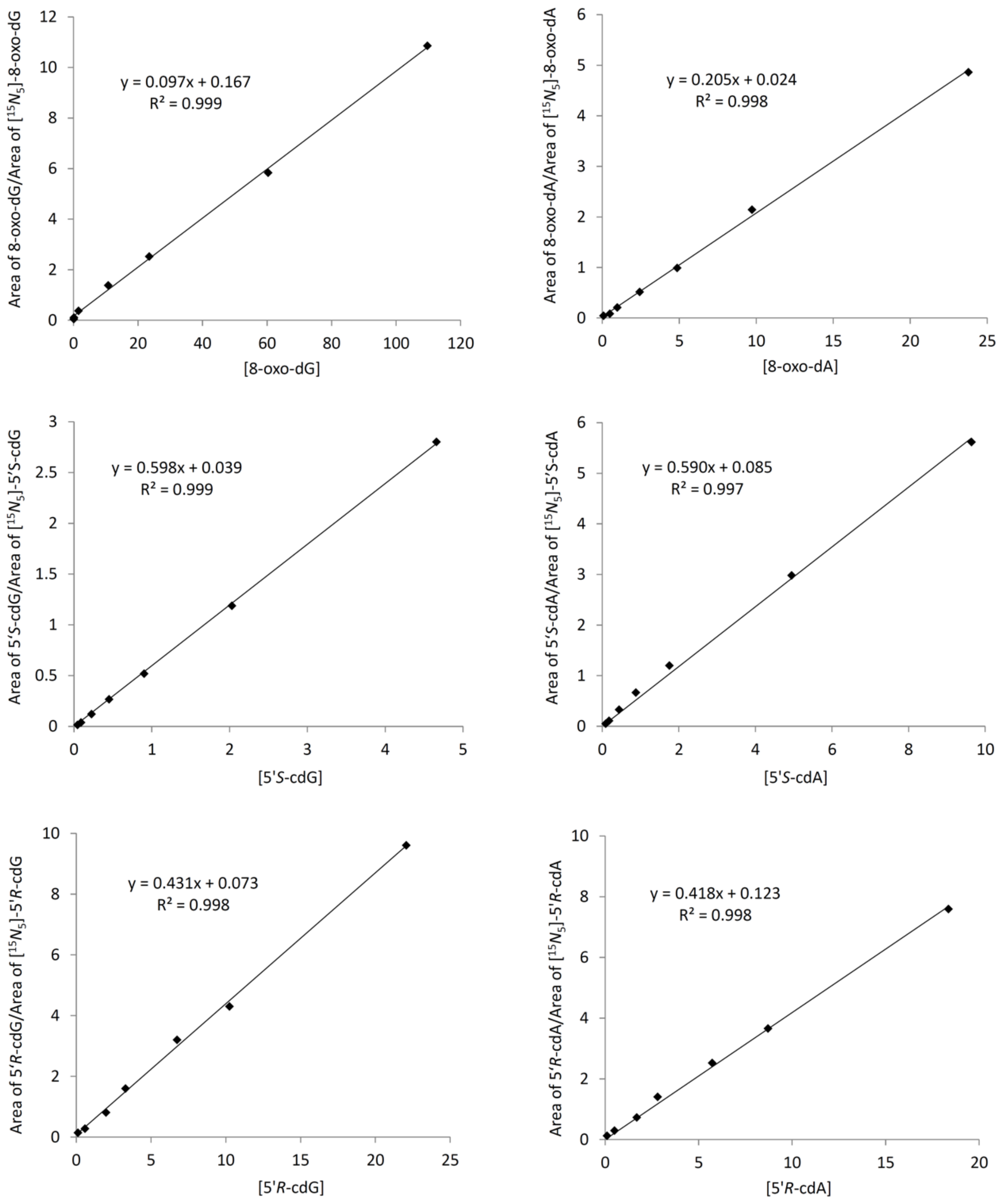

Figure S3. Calibration curves for the quantification of the lesions (nM)

Table S1. A list of MRM transitions employed for the quantifications of the six oxidatively induced DNA lesions and their corresponding stable isotope-labeled standards.

Lesions	Precursor ion m/z	Product ion m/z	Collision energy (V)
5'*R*-cdA	250	164	14
[$^{15}N_5$]-5'*R*-cdA	255	169	14
5'*S*-cdA	250	164	16
[$^{15}N_5$]- 5'*S*-cdA	255	169	16
5'*R*-cdG	266	180	18
[$^{15}N_5$]- 5'*R*-cdG	271	185	18
5'*S*-cdG	266	180	16
[$^{15}N_5$]- 5'*S*-cdG	271	185	16
8-oxo-dA	267	151	19
[$^{15}N_5$]- 8-oxo-dA	272	156	19
8-oxo-dG	284	168	18
[$^{15}N_5$]- 8-oxo-dG	289	173	18

Table S2: Total amount of 5'*R*-cdG, 5'*S*-cdG, 5'*R*-cdA, 5'*S*-cdA, 8-oxo-dG and 8-oxo-dA (lesions/10^7 nucleosides) in DNA applying Procedure (i). The numbers in the boxes represent the mean value (± standard deviation) of DNA lesions levels from the measurement of three samples.

Dose, Gy	5'*R*-cdG	5'*R*-cdA	5'*S*-cdG	5'*S*-cdA	8-oxo-dG	8-oxo-dA
0	0.4±0.01	0.2±0.01	0.04±0.01	0.7±0.25	13.3±0.7	2.3±0.8
10	10.8±5.3	15.2±6.1	3.4±0.7	11.5±4.5	2128.1±322.5	134.4±16.6
20	24.5±10.6	25.2±8.2	5.5±1.6	25.2±6.5	4542.7±1503.4	341.3±80.6
35	56.6±16.1	50.9±13.9	10.1±1.7	39.7±9.5	6893.4±2272.6	627.7±117.5
50	69.5±20.4	80.2±11.5	16.1±2.6	69.0±4.9	9044.2±1346.6	1106.7±163.9

Table S3: Total amount of 5'*R*-cdG, 5'*S*-cdG, 5'*R*-cdA, 5'*S*-cdA, 8-oxo-dG and 8-oxo-dA (lesions/10^7 nucleosides) in DNA applying Procedure (ii). The numbers in the boxes represent the mean value (± standard deviation) of DNA lesions levels from the measurement of three samples.

Dose, Gy	5'*R*-cdG	5'*R*-cdA	5'*S*-cdG	5'*S*-cdA	8-oxo-dG	8-oxo-dA
0	0.4±0.01	0.2±0.01	0.04±0.01	0.7±0.25	14.7±0.6	2.9±0.7
10	10.6±6.5	14.4±5.3	3.2±0.6	10.9±3.4	1779.1±445.6	142.9±47.1
20	31.3±10.1	28.4±10.0	7.3±2.3	29.2±10.9	4436.9±1660.2	408.6±110.8
35	57.7±10.7	54.3±11.3	12.9±2.3	42.2±5.2	6811.2±1067.1	727.0±204.7
50	61.1±16.4	75.6±13.3	15.1±2.0	64.8±4.6	8126.7±880.5	1096.6±226.4

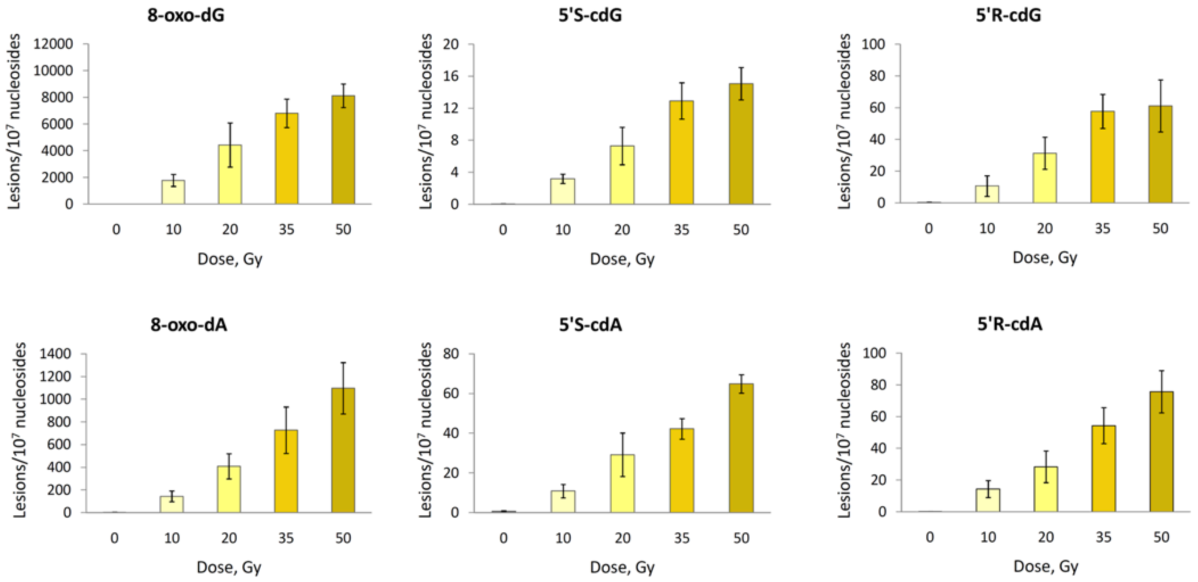

Figure S4. Radiation induced formation of 8-oxo-dG, 5'*S*-cdG, 5'*R*-cdG, 8-oxo-dA, 5'*S*-cdA and 5'*R*-cdA in ct-DNA Procedure (ii). Each sample was exposed to 0 Gy, 10 Gy, 20 Gy, 35 Gy and 50 Gy dose. The values represent the mean ± SD of *n*=3 independent experiments.

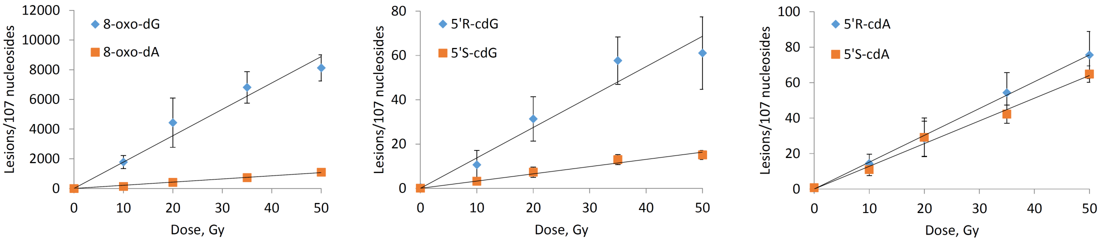

Figure S5. Radiation induced formation of 8-oxo-dG, 8-oxo-dA, 5'R-cdG, 5'S-cdG, 5'R-cdA, and 5'S-cdA in ct-DNA; Procedure (ii). Each sample was exposed to 0, 10, 20, 35 and 50 Gy dose in N2O-saturated aqueous solutions; The values represent the mean ± SD of n=3 independent experiments.

Table S4: The levels (lesions/10^7 nu/Gy) of 8-oxo-dG, 8-oxo-dA, 5'*R*-cdA, 5'*S*-cdA, 5'*R*-cdG and 5'*S*-cdG in calf thymus DNA.

Lesions	Procedure (i) lesions/10^7 nu/Gy	Procedure (ii) lesions/10^7 nu/Gy
8-oxo-dG	177.1 ± 20.6	171.8 ± 13.0
8-oxo-dA	22.3 ± 1.7	22.3 ± 1.9
5'*R*-cdA	1.58 ± 0.13	1.53 ± 0.12
5'*S*-cdA	1.33 ± 0.09	1.27 ± 0.08
5'*R*-cdG	1.48 ± 0.17	1.33 ± 0.16
5'*S*-cdG	0.31 ± 0.02	0.31 ± 0.03

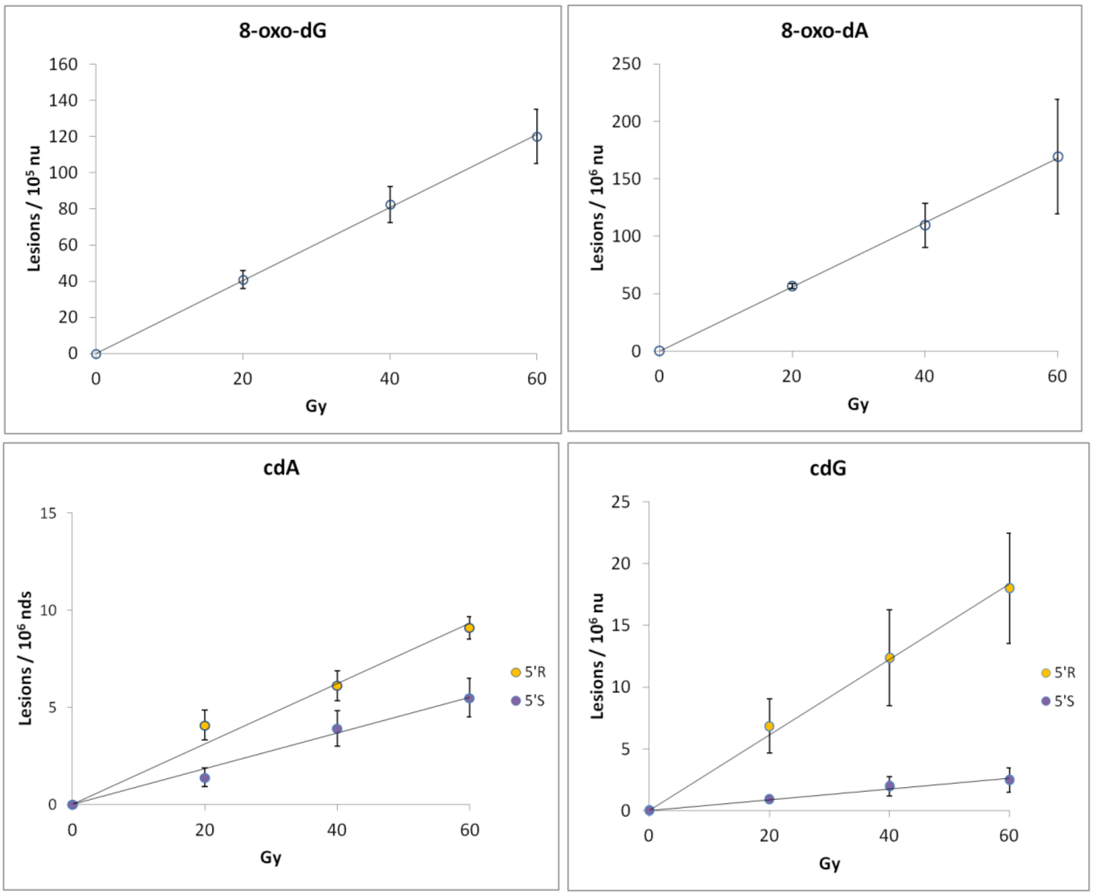

Figure S6: Radiation induced formation of 8-oxo-dG, 8-oxo-dA, (5'R ●, 5'S ●) and cdA (5'R ●, 5'S ●) in aqueous N_2O saturated of ct-DNA. Each data point represents the mean of n=3 independent experiments and the uncertainties are the standard errors. The values obtained from each independent experiment were normalized by subtracting the background levels of the lesions (Taken from Ref. [33]).

Table S5. Sequences and molecular masses of the synthesized ODNs

Strands	Sequence (5'- 3')	Mass calcd (Da)	Mass[a] found (Da)
**ODN-5**	GGG (TTA GGG)$_3$	6653.40	6653.40
**ODN-6[b]**	CCC(TAA CCC)$_3$	6200.10	6199.40

[a]All the oligonucleotide masses were obtained by ESI in negative mode. [b] Complementary strand

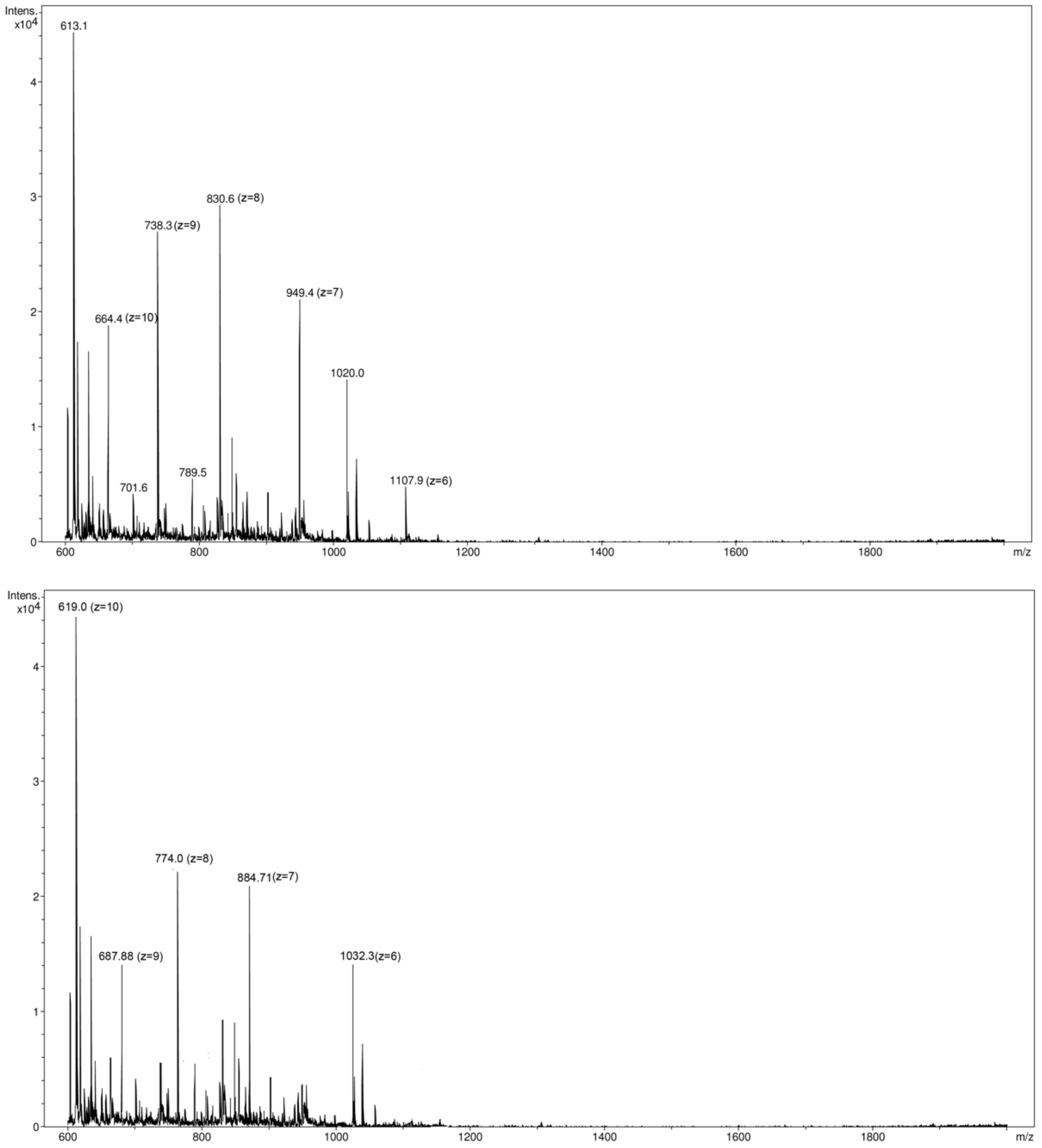

Figure S7. ESI spectra of ODN5 (upper part) and ODN6 (lower part). ESI spectra of DNA strands were measured in flow injection 5 μL sample (25 μM DNA, 2.5 mM diammonium citrate) were injected in a steady flow of H_2O:ACN; 90:10 (200 μL/min). The capillarity temperature was 300°C, spray voltage 3.5 KV (negative mode).

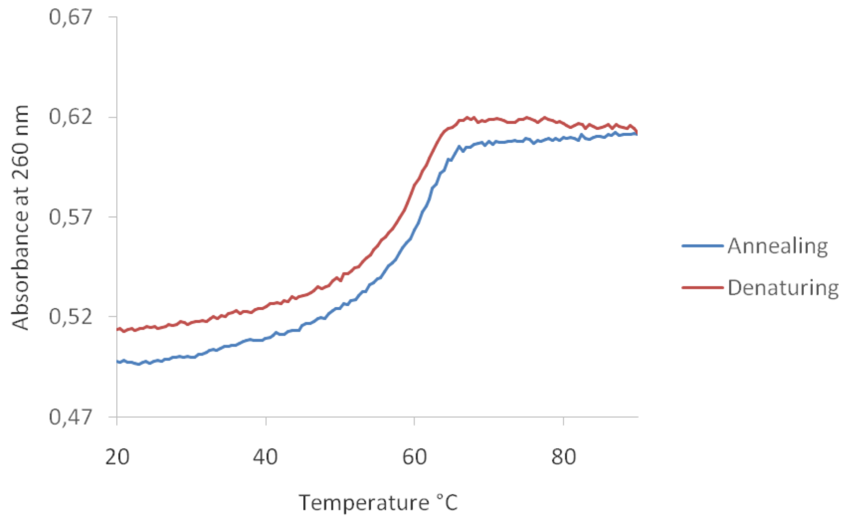

Figure S8. UV melting curves of 21-mer duplexes. The oligonucleotide strand 5′-d[GGG(TTA GGG)$_3$]-3 (ODN-5), was annealed to the complementary strand in equimolar concentration in buffer solution containing 50 mM sodium phosphate, pH 7.2. The substrate was constructed by heating the two strands at 90°C for 10 min and subsequently allowing the temperature to slowly drop down to the room temperature (25 °C). Melting temperature (Tm) of the substrate was measured with a Cary 100 UV/Vis spectrometer using a 1 mL quartz cuvette with a 1 cm pathlenght. This allowed to monitor the absorbance of the solutions at 260 nm as a function of temperature. The temperature cycle was recorded from 20 to 90 °C with a temperature controller at a heating rate of 0.3°C/min. **Tm** = 60°C

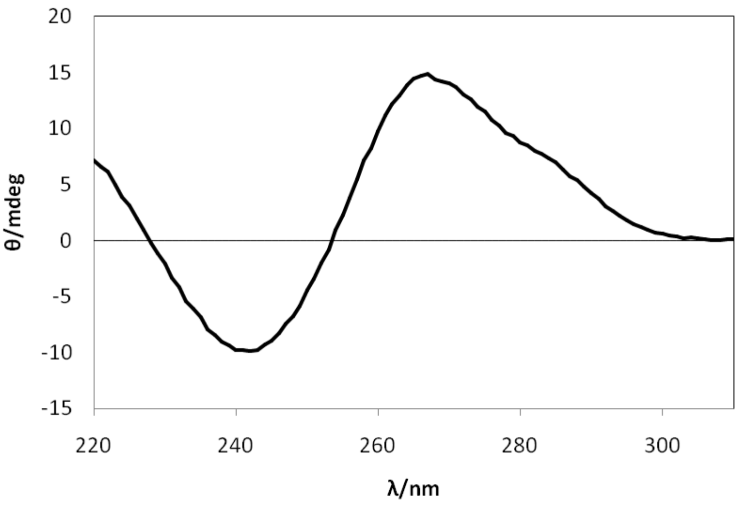

Figure S9. CD spectra were recorded on a *Jasco J-710* spectropolarimeter using a quartz cuvette (0.1 cm optical path length) at a scanning speed of 50 nm/min with 1 s response time. Measurements at the range of 200-360 nm were the average of four accumulations at 295 K and smoothed with Origin, Version 8.00 program. The sample contained an aqueous solution of 50 mM sodium phosphate, pH 7.2 and 50 μM double stranded oligonucleotide substrates. The reported spectrum was obtained by subtracting the spectrum of blank (aqueous solution of sodium phosphate buffer).

Table S6. Total amount of 8-oxo-dG, 8-oxo-dA, 5'*S*-cdG, 5'*S*-cdA, 5'*R*-cdG and 5'*R*-cdA (lesions/10^7 dG or dA) in double-stranded 21-mer oligonucleotides. Each sample was exposed to 0, 20, 40 and 60 Gy dose in N_2O-saturated aqueous solutions; The values represent the mean value ± SD of n=3 independent experiments.

Lesions	0 Gy	20 Gy	40 Gy	60 Gy
8-oxo-dG	0.0 ± 0.0	239.3 ± 74.9	623.3 ± 145.9	776.0 ± 201.9
8-oxo-dA	0.0 ± 0.0	48.3 ± 1.5	73.1 ± 12.7	124.9 ± 41.1
5'*S*-cdG	0.0 ± 0.0	9.8 ± 1.2	27.1 ± 6.7	51.1 ± 11.5
5'*S*-cdA	0.0 ± 0.0	29.2 ± 14.7	43.3 ± 9.6	96.5 ± 10.1
5'*R*-cdG	0.0 ± 0.0	6.1 ± 2.1	11.4 ± 4.0	22.0 ± 3.4
5'*R*-cdA	0.0 ± 0.0	36.5 ± 5.9	51.3 ± 16.6	102.3 ± 18.2

Table S7. The levels (lesions/10^6 dG or 10^6 dA) of 8-oxo-dG, 8-oxo-dA, 5'*R*-cdG, 5'*S*-cdG, 5'*R*-cdA and 5'*S*-cdA upon Fenton-type reagent treatment of ds-ODNs. The numbers in the boxes represent the values of DNA lesions levels from the measurement of each sample.

$CuCl_2$ (μM)/H_2O_2 (μM)/Ascorbate (mM)	8-oxo-dG	8-oxo-dA	5'*R*-cdG	5'*S*-cdG	5'*R*-cdA	5'*S*-cdA
5/40/0.4	65.96	14.92	2.70	2.90	3.43	2.17
5/40/0.4	58.97	14.35	1.86	2.58	3.17	2.61
5/40/0.4	63.24	26.52	2.59	3.02	4.52	3.04
10/80/0.8	12.78	22.49	5.50	8.90	6.19	5.20
10/80/0.8	110.75	21.79	6.60	7.82	8.78	4.86
10/80/0.8	102.70	17.13	6.28	8.87	8.60	4.35
15/120/1.2	202.20	33.33	9.75	13.49	14.50	8.92
15/120/1.2	265.77	38.09	10.73	14.15	14.03	8.42
15/120/1.2	237.75	38.71	9.79	13.05	13.31	9.24

Table S8. Total amount of 8-oxo-purine lesions and cPu lesions (lesions/10^6 dG or 10^6 dA) upon Fenton-type reagent treatment of ds-ODNs. The numbers in the boxes represent the mean value (± standard deviation) of DNA lesions levels from the measurement of three samples.

$CuCl_2$ (μM)/H_2O_2 (μM)/Ascorbate (mM)	8-oxo-dG	8-oxo-dA	5'*R*-cdG	5'*S*-cdG	5'*R*-cdA	5'*S*-cdA
5/40/0.4	62.72±3.52	18.60±6.87	2.38±0.46	2.83±0.23	3.71±0.72	2.60±0.43
10/80/0.8	113.74±12.80	20.47±2.92	6.13±0.57	8.53±0.61	7.86±1.45	4.80±0.43
15/120/1.2	235.24±31.86	36.71±2.94	10.09±0.56	13.56±0.55	13.95±0.60	8.86±0.41

Table S9. The diastereoisomeric ratios (5'*R*/5'*S*) for both cdG and cdA upon Fenton-type reagent treatment of ds-ODNs and. The numbers in the boxes represent the mean value (± standard deviation) of DNA lesions levels from the measurement of three samples.

$CuCl_2$ (μM)/H_2O_2 (μM)/Ascorbate (mM)	5′*R*/5′*S* ratio for cdG	5′*R*/5′*S* ratio for cdA
5/40/0.4	0.84±0.11	1.43±0.19
10/80/0.8	0.72±0.11	1.66±0.41
15/120/1.2	0.74±0.02	1.58±0.12

Table S10. The levels (lesions/10^6 dG or 10^6 dA) of 8-oxo-dG, 8-oxo-dA, 5'*R*-cdG, 5'*S*-cdG, 5'*R*-cdA and 5'*S*-cdA upon treatment of ds-ODNs with $CuCl_2$ (15 uM), H_2O_2 (120 uM), Ascorbate (1.2 mM) at 20 min, 40 min, 60 min, 90 min and 120 min. The numbers in the boxes represent the values of DNA lesions levels from the measurement of each sample.

time (min)	8-oxo-dG	8-oxo-dA	5'R-cdG	5'S-cdG	5'R-cdA	5'S-cdA
20	98.20	14.29	4.70	5.57	8.06	5.83
20	74.70	20.60	6.00	6.96	7.91	3.48
40	187.14	19.46	7.39	9.51	11.95	6.32
40	112.17	27.95	7.34	10.50	8.36	5.17
60	283.86	23.38	11.56	14.31	13.01	6.96
60	223.60	40.76	11.29	15.81	12.10	10.65
90	399.59	55.17	10.72	15.19	19.64	18.10
90	290.21	47.19	14.27	14.08	19.14	13.13
120	984.60	82.39	22.70	20.58	27.80	17,58
120	643.22	70.47	19.75	26.06	23.46	15.14

Table S11. Total amount of 8-oxo-purine lesions and cPu lesions (lesions/10^6 dG or 10^6 dA) formation upon treatment of ds-ODNs with $CuCl_2$ (15 uM), H_2O_2 (120 uM), Ascorbate (1.2 mM) at 20 min, 40 min, 60 min, 90 min and 120 min.The numbers in the boxes represent the mean value (± standard deviation) of DNA lesions levels from the measurement of two samples.

Time (min)	8-oxo-dG	8-oxo-dA	5'R-cdG	5'S-cdG	5'R-cdA	5'S-cdA
20	86.45±16.62	17.45±4.46	5.35±0.92	6.26±0.98	7.99±0.11	4.65±1.66
40	149.66±53.01	23.71±6.00	7.37±0.03	10.00±0.71	10.16±2.54	5.75±0.82
60	253.73±42.60	32.07±12.29	11.42±0.19	15.06±1.06	12.55±0.64	8.81±2.62
90	344.90±77.34	51.18±5.64	12.49±2.51	14.64±0.79	19.39±0.35	15.61±3.51
120	813.91±241.39	76.43±8.43	21.23±2.09	23.32±3.88	25.63±3.07	16.36±1.73

Table S12: The diastereoisomeric ratios (5'*R*/5'*S*) for both cdG and cdA upon treatment of ds-ODNs with $CuCl_2$ (15 uM), H_2O_2 (120 uM), Ascorbate (1.2 mM) at 20, 40, 60, 90 and 120 min. The numbers in the boxes represent the mean value (± standard deviation) of DNA lesions levels from the measurement of two samples.

Time (min)	5′*R*/5′*S* ratio for cdG	5′*R*/5′*S* ratio for cdA
20	0.85±0.01	1.83±0.63
40	0.74±0.01	1.75±0.19
60	0.76±0.07	1.50±0.52
90	0.86±0.22	1.27±0.26
120	0.91±0.24	1.57±0.02

Table S13. The levels (lesions/10^6 dG or 10^6 dA) of 8-oxo-dG, 8-oxo-dA, 5'*R*-cdG, 5'*S*-cdG, 5'*R*-cdA and 5'*S*-cdA upon treatment of ds-ODNs with Fe^{2+} (15 uM), H_2O_2 (120 uM), Ascorbate (1.2 mM) at 30, 60, 90 and 120 min. The numbers in the boxes represent the values of DNA lesions levels from the measurement of each sample.

Time (min)	8-oxo-dG	8-oxo-dA	5'R-cdG	5'S-cdG	5'R-cdA	5'S-cdA
30	113.01	23.03	5.08	6.15	12.00	6.89
30	110.16	14.14	5.32	6.66	7.63	5.94
60	214.29	42.85	8.84	16.00	13.22	12.00
60	309.74	29.36	8.96	18.18	15.78	8.69
90	364.47	35.67	14.34	19.66	19.52	15.06
90	496.66	60.24	10.21	19.49	21.09	12.19
120	965.48	51.31	22.98	26.05	26.22	17.95
120	978.92	65.89	23.77	25.42	26.34	18.20

Table S14. Total amount of 8-oxo-purine lesions and cPu lesions (lesions/10^6 dG or 10^6 dA) formation upon treatment of ds-ODNs with Fe^{2+} (15 uM), H_2O_2 (120 uM), Ascorbate (1.2 mM) at at 30, 60, 90 and 120 min. The numbers in the boxes represent the mean value (± standard deviation) of DNA lesions levels from the measurement of two samples.

Time (min)	8-oxo-dG	8-oxo-dA	5'R-cdG	5'S-cdG	5'R-cdA	5'S-cdA
**30**	111.58±2.01	18.58±6.29	5.20±0.17	6.41±0.37	9.82±3.10	6.41±0.67
**60**	262.01±67.50	36.10±9.54	8.90±0.09	17.09±1.54	14.50±1.81	10.34±2.34
**90**	430.57±93.47	47.95±17.38	12.28±2.92	19.58±0.12	20.31±1.11	13.62±2.03
**120**	972.20±9.50	58.60±10.31	23.38±0.56	25.73±0.44	26.28±0.09	1.08±0.18

Table S15. The diastereoisomeric ratios (5'*R*/5'*S*) for both cdG and cdA upon treatment of ds-ODNs with Fe^{2+} (15 uM), H_2O_2 (120 uM), Ascorbate (1.2 mM) at 30, 60, 90 and 120 min. The numbers in the boxes represent the mean value (± standard deviation) of DNA lesions levels from the measurement of two samples.

Time (min)	5′*R*/5′*S* ratio for cdG	5′*R*/5′*S* ratio for cdA
30	0.81±0.02	1.51±0.33
60	0.52±0.04	1.46±0.50
90	0.63±0.15	1.51±0.31
120	0.91±0.04	1.45±0.01
